# Antimalarial drug resistance and population structure of *Plasmodium falciparum* in Mozambique using genomic surveillance at health facilities in 2021 and 2022

**DOI:** 10.1038/s41598-025-02166-w

**Published:** 2025-08-11

**Authors:** Simone Boene, Eduard Rovira-Vallbona, Clemente da Silva, Manuel García-Ulloa, Bernardete Rafael, Neide Canana, Andrés Aranda-Díaz, Pau Cisteró, Carla García-Fernández, Dário Tembisse, Nelo Ndimande, Arlindo Chidimatembue, Glória Matambisso, Brian Palmer, Ana Rita Chico, Mércia Dimene, Abuchahama Saifodine, José Inácio, Mariana da Silva, Mateusz Plucinski, Craig Bonnington, Flavio Wate, Eva de Carvalho, Guidion Mathe, Arnau Pujol, Beatriz Arregui-Gallego, Kiba Comiche, Abel Nhama, Lídia Nhamussua, Pedro Aide, Francisco Saute, Sónia Enosse, Bryan Greenhouse, Baltazar Candrinho, Alfredo Mayor

**Affiliations:** 1 Centro de Investigação em Saúde de Manhiça (CISM), Maputo, Mozambique; 2 ISGlobal, Barcelona, Spain; 3 Facultat de Medicina i Ciències de la Salut, Universitat de Barcelona (UB), Barcelona, Spain; 4 National Malaria Control Program, Ministry of Health, Maputo, Mozambique; 5 Malaria Consortium, London, UK; 6 EPPIcenter Research Program, Division of HIV, Infectious Disease and Global Medicine, Department of Medicine, University of California, San Francisco, San Francisco, USA; 7 Clinton Health Access Initiative, Maputo, Mozambique; 8 President’s Malaria Initiative, USAID, Maputo, Mozambique; 9 Malaria Branch, Centers for Disease Control and Prevention, Atlanta, GA USA; 10 World Health Organization, Maputo, Mozambique; 11 Faculdade de Medicina, Universidade Eduardo Mondlane, Maputo, Mozambique; 12 Spanish Consortium for Research in Epidemiology and Public Health (CIBERESP), Madrid, Spain; 13 Instituto Nacional de Saúde (INS), Marracuene, Mozambique

**Keywords:** *Plasmodium falciparum*, Malaria, Genomics, Surveillance, Drug resistance, Artemisinin, Genetic diversity, Mozambique, Biotechnology, Computational biology and bioinformatics, Genetics, Malaria

## Abstract

Monitoring the emergence and spread of drug-resistant parasites is essential for effective malaria control. Here, we describe the prevalence of genetic markers of *Plasmodium falciparum* antimalarial drug resistance and parasite population structure in Mozambique. Drug resistance loci and microhaplotypes were genotyped by multiplex targeted amplicon sequencing of 1146 *P. falciparum* samples collected in 2021 (*n* = 321) and 2022 (*n* = 825 rainy season, and *n* = 155 dry season). *pfpm2* gene copy number (associated to piperaquine resistance) was assessed using real-time quantitative PCR. No *pfk13* markers of partial artemisinin resistance nor *pfpm2* duplications were observed. Prevalence of *pfdhfr/pfdhps* quintuple mutants associated with sulfadoxine-pyrimethamine (SP) resistance was high across all regions (> 92.5% in 2021 and > 87.8% in 2022), but *pfdhps*-A581G mutation was rare (1.6% in 2021 and 0.8% 2022). Both prevalence of mutations in *pfdhps*-436 (*p* < 0.001) and genetic complexity of infections increased from South to North. These results support the continued use of artemisinin-based combination therapies in Mozambique, call for a close monitoring of chemopreventive efficacy based on SP, and confirm the spatial genetic distinction in *P. falciparum* population observed across the country.

**Supplementary Information:**

The online version contains supplementary material available at 10.1038/s41598-025-02166-w.

## Introduction

Mozambique is one of the four African countries that accounts for over half of all malaria deaths worldwide, the vast majority attributable to *Plasmodium falciparum*^1^. Malaria transmission is highly heterogeneous across the country^2,3^, requiring different strategies to both reduce the burden of malaria where the transmission is high (northern and central Mozambique), and to eliminate malaria where the transmission is low (southern Mozambique). Pharmacological-based interventions are one of the main pillars of National Malaria Control Program (NMCP) approaches to malaria control and elimination across transmission strata^4^. These include the effective treatment of malaria clinical cases using artemisinin-based combination therapy (ACT; first line treatments: artemether-lumefantrine [AL] and artesunate-amodiaquine [AS-AQ])^5^, chemoprevention in children (i.e., perennial malaria chemoprevention [PMC] with sulfadoxine-pyrimethamine [SP] and seasonal malaria chemoprevention [SMC] with SP-AQ), chemoprevention in pregnant women (i.e., intermittent preventive treatment [IPTp] with SP), and treatment of the whole population in near elimination or emergency settings (mass drug administration [MDA] with dihydroartemisinin-piperaquine [DP])^5^.

*P. falciparum* has shown a remarkable ability to develop resistance to multiple antimalarial drugs, and the emergence of resistance to artemisinin and partner drugs within ACTs in Africa poses a significant threat for their potential impact on antimalarial interventions^6-11^. Monitoring the emergence, evolution and spread of drug-resistant parasites is crucial for implementing effective malaria control strategies. The gold standard for drug efficacy, the in vivo therapeutic efficacy studies (TES), are logistically challenging and costly, thus conducted intermittently and at a limited number of sites, potentially missing geographical heterogeneities. Genomic surveillance constitutes a powerful approach for high-throughput and routine data generation that can complement and/or inform TES, as it has the potential to provide information not only on a wide range of resistance genotypes but also on other genotypes of programmatic interest, such as vaccine targets or markers of genetic diversity for TES case classification^12^, parasite importation or transmission intensity stratification.

Several genetic polymorphisms have been validated as markers of antimalarial resistance and associated with failure to ACT treatment^13^. Point mutations in *kelch 13* gene (*pfkelch13*) confer partial resistance to artemisinin derivatives^14^, and multiple gene copies in *plasmepsin-2* (*pfpm2*) have been associated with resistance to the piperaquine, the partner drug in DP formulation^15^. However, there is no well-validated marker of resistance for lumefantrine in AL, which is currently the most common treatment in both public and private sector markets in Africa^13^. In the case of SP, parasitological resistance can be conferred by accumulation of mutations in the genes *dihydrofolate reductase* (*pfdhfr;* codons 16, 51, 59, 108, 164) and *dihydropteroate synthase* (*pfdhps*; codons 431, 436, 437, 540, 581, 613). The quintuple combination *pfdhfr* 51-59-108 + *pfdhps* 437–540 and, most notably, the sextuple combination that includes *pfdhps*-581 have been associated to clinical and parasitological failures to SP treatment^16,17^. Resistance to chloroquine is conferred by mutation 76 in *chloroquine transporter* gene *(pfcrt*), whereas point mutations in multidrug resistance gene 1 *(pfmdr1)* in combination with *pfcrt* mutants have been associated to amodiaquine resistance^18^. To date, only one study has reported the spatial and temporal distribution of antimalarial resistance in Mozambique with data from 2018^19^. This study indicated that parasite population was characterized by a south-to-north decreasing gradient in resistance to SP (i.e., prevalence of quintuple mutants), accompanied by a concentration of *pfdhps*-436 mutations (A/C/F/H) in the North, and south-to-north increases in genetic complexity, pointing to the importance of understanding geographical heterogeneity of *P. falciparum* population, its relation with transmission intensity, and its potential impact on policy.

Since 2021, Mozambique’s NMCP has been relying on the genomic surveillance for guiding programmatic decisions^20^. Among priority use cases are (1) the routine monitoring of drug and diagnostic resistance markers and (2) characterization of genetic diversity metrics across different malaria transmission intensity strata to inform malaria burden (i.e. as complementary indicators of epidemiological metrics of transmission intensity)^20,21^. For this purpose, a novel multiplex amplicon sequencing assay with capacity to generate data for 15 drug-resistance genes and 165 highly diverse microhaplotypes was established^22^. Here we present the first report of this surveillance effort with high throughput sequencing data generated in-country and investigate parasite population structure at different regions as well as the effect of seasonality on genetic metrics.

## Results

### Sample selection and geographical coverage during 2021 and 2022 rainy seasons

**Fig. 1:**
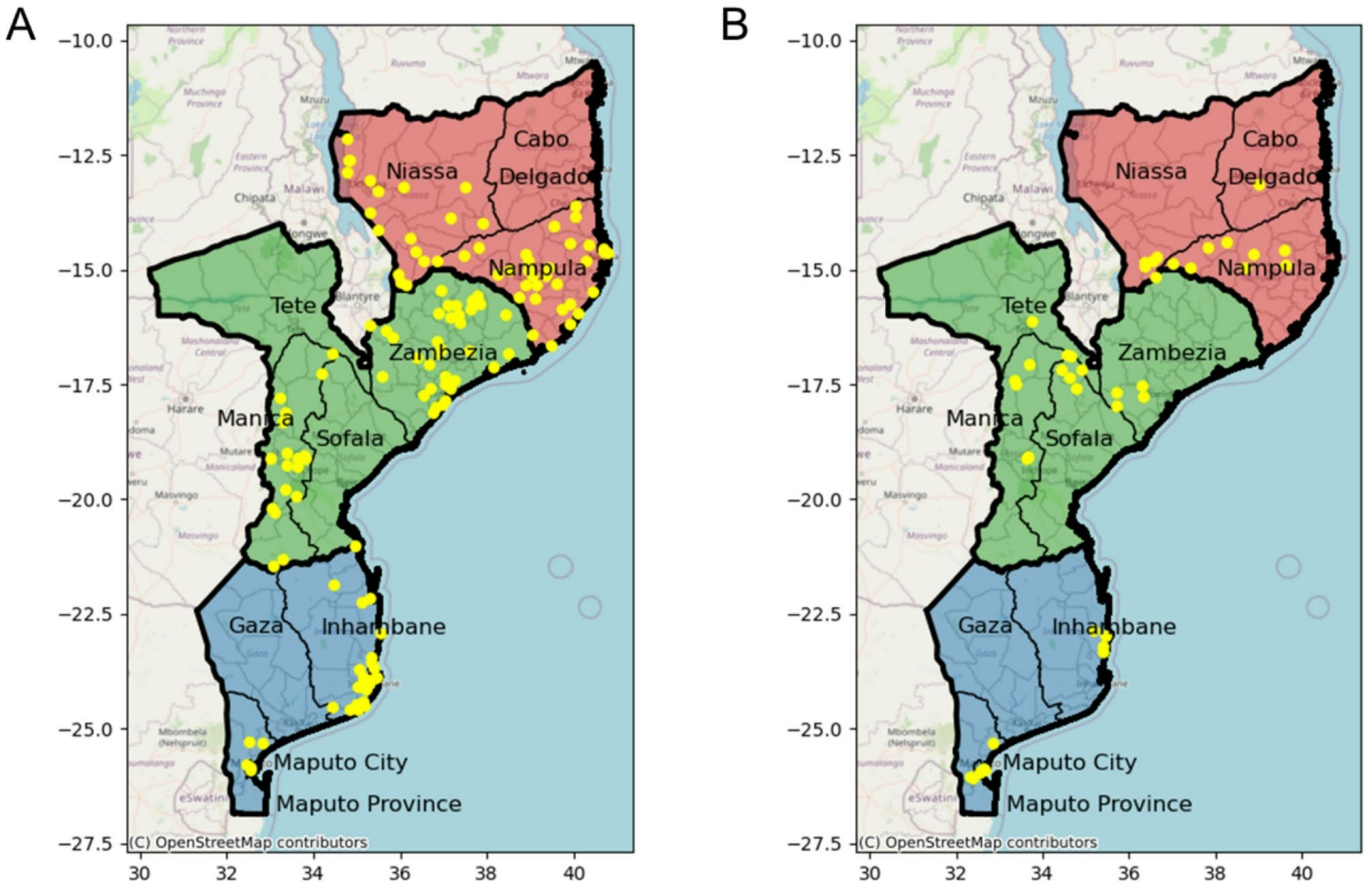
Location of health facilities included in the study. Map of Mozambique with province (thick black lines) and district (thin grey lines) administrative divisions. Yellow dots indicate the location of health facilities in the 2021 (A) and 2022 (B) surveys. Geographical regions used in primary analysis are colored (South in blue, Centre in green, North in red). Map generated from OpenStreetMap data available under the Open Database License and modified in Python 3.

Patients with a confirmed diagnosis of uncomplicated *P. falciparum* malaria by rapid diagnostic test (RDT) were recruited at different cross-sectional surveys at health facility level during two consecutive rainy seasons (January to May) in 2021 and 2022. One hundred dried blood spot (DBS) samples were randomly selected for sequencing per province and year, unless the sample size was less than 100, resulting in a total of 1285 DBS (387 from 2021 to 898 from 2022). The locations where the samples were collected are presented in Fig. 1. Provinces with initial number of DBS < 100 were Inhambane (80), Manica (33) and Niassa (67) in 2021, and Sofala (86) and Cabo Delgado (91) in 2022. Positive qPCR confirmation followed by successful sequencing (i.e., with allele calls passing negative controls and allele frequency filters, see Methods) was obtained from a total of 321 DBS for 2021 (83%) and 825 (92%) from 2022, as detailed in Table 1. With sample size computed at the regional level (South, Center and North), the study had a > 80% power for the detection of genetic variants at a frequency of 2% for 2021, and 1% for 2022^23^.

Demographic characteristics of patients and parasite densities determined by *P. falciparum* 18S qPCR are shown in Table 1. Age differed between regions, with a higher proportion of older than five-year patients in the South compared to Centre and North. Note that children were the targeted population in all surveys, except in Maputo Province (in the South) in 2022, which also included adults. Patients did not differ in gender (*p* > 0.116). Overall parasite densities did not differ between 2021 and 2022 (*p* = 0.305). Between regions, densities were significantly higher in the South only in 2021 (*p* < 0.001; Table 1).

**Table 1:** Number of samples sequenced, demographic characteristics of study participants and parasitemia, by year and geographical region.

	2021					2022				
	All regions	South	Center	North	*p*-value	All regions	South	Center	North	*p*-value
N	321	73	111	137		825	191	353	281	
Province, n (%)										
Cabo Delgado	-	-	-	-	-	91 (11.0%)	-	-	91 (32.4%)	-
Niassa	54 (16.8%)	-	-	54 (39.4%)	-	92 (11.2%)	-	-	92 (32.7%)	-
Nampula	83 (25.8%)	-	-	83 (60.6%)	-	98 (11.9%)	-	-	98 (34.9%)	-
Zambezia	85 (26.5%)	-	85 (76.6%)	-	-	88 (10.7%)	-	88 (24.9%)	-	-
Tete	-	-	-	-	-	98 (11.9%)	-	98 (27.8%)	-	-
Sofala	-	-	-	-	-	86 (10.4%)	-	86 (24.4%)	-	-
Manica	26 (8.1%)	-	26 (23.4%)	-	-	81 (9.8%)	-	81 (22.9%)	-	-
Inhambane	68 (2.1%)	68 (93.2%)	-	-	-	91 (11.0%)	91 (47.6%)	-	-	-
Maputo	5 (1.6%)	5 (6.8%)	-	-	-	100 (12.1%)	100 (52.4%)	-	-	-
Female, n (%)	160 (48.8%)	32 (43.8%)	64 (57.7%)	64 (46.7%)	0.116	396 (48.7%)	95 (51.6%)	170 (48.9%)	131 (47.7%)	0.570
Age:										
Years, median (IQR)	4 (2, 6)	6 (4, 8)	3 (2, 6)	4 (2, 6)	< 0.001	4 (2, 7)	8 (5, 16)	4 (3, 6)	2 (1, 4)	< 0.001
< 5 years, n (%)	175 (54.5%)	25 (34.2%)	71 (64%)	79 (0.577%)	< 0.001	499 (61.2%)	51 (27.7%)	220 (62.9%)	228 (81.1%)	< 0.001
≥ 5 years, n (%)	146 (45.5%)	48 (65.8%)	40 (36%)	58 (0.423%)		316 (38.8%)	133 (72.3%)	130 (37.1%)	53 (18.9%)	
Parasitemia, median parasites/µL (IQR)	10,209 (355, 66613)	51,278 (6773, 116619)	4169 (194, 38332)	8392 (391, 62381)	< 0.001	14,788 (1386, 52473)	17,863 (2888, 48929)	17,528 (1604, 56330)	9746 (543, 53204)	0.165

## Genetic markers of drug resistance

Extracted DNA was subject to amplicon-based sequencing using the MAD4HatTeR modular assay^22^. The following drug resistance genes were analyzed for genetic polymorphisms, based on current, past or planned uses of antimalarials in Mozambique: *pfk13*,* pfdhps*,* pfdhfr*,* pfcrt*,* pfmdr1* and *pfpm2.* In addition, *pfexonuclease*,* pfarps10*,* pfferredoxin*,* pfmdr2* were screened for their potential role in artemisinin and DP resistance. The sequencing depth for the amplicons covering selected loci was 1155 median reads (interquartile range, IQR: 592, 2086). By study year, the median was 653 reads/sample/amplicon (IQR 352, 1025) in 2021 samples, and 1499 reads/sample/amplicon (IQR 807, 2512) in 2022 samples (**Supplementary Fig. 1**).

### Polymorphisms in *pfk13* gene and artemisinin-resistance background

The prevalence of infections carrying mutant parasites by year and geographical region is presented in Table 2 (detail by province in **Supplementary Tables 1 and 2**). None of the samples sequenced from the 2021 or 2022 rainy seasons carried *pfk13* validated or candidate markers of partial artemisinin resistance. However, up to 17 different non-synonymous mutations were found in 20 different samples, accounting for 2% (6/297) of infections in 2021 and 1.8% (14/772) in 2022 (Tables 2 and 3). Seventeen out of the 20 non-synonymous mutants were part of mixed infections with the wild-type allele. A higher prevalence of non-synonymous mutations was detected in the North (3.1–3.2%) as compared to Centre (0.9-2%) and South (0-1.1%), but the trend was not statistically significant. Mutations G449S (South, Maputo province, 2022) and R561C (North, Nampula province, 2022) occurred at the same codon as other validated/associated mutations^13^. Mutations associated with a parasite genetic background favorable for the emergence of artemisinin resistance in South-East Asia, namely, *pfarps10* (V127M), *pfferredoxin* (D193Y) and *pfmdr2 (*T484I)^24^, were not present in Mozambique.

### Polymorphisms in *pfpm2* and other piperaquine-resistance associated markers

Read counts were used to screen for candidate samples carrying putative *pfpm2* copy number variations (CNV, defined as > 1.5-fold deviations from the expected read counts for single-copy *pfpm2*), a genotype associated with resistance to the partner drug piperaquine in DP formulation^25^. The screening was conducted in samples with parasitemia > 1000 parasites/µl to enhance robustness (74,6%, corresponding to 855 out of 1146; see Methods). Twenty-three (2.6%) samples had an observed fold-change > 1.5 (median 1.6 [IQR 1.5, 1.8]), but none was confirmed to carry CNV in subsequent qPCR analysis (see Methods). Mutation E415G in *pfexonuclease*, previously linked to reduced piperaquine IC50 in genome-wide association analysis^26^, and mutations T93S, H97Y, F145I, I218F and C350R in *pfcrt*, associated to minor-to-moderate decreases in in vivo or in vitro piperaquine resistance outside Africa^27^, were not detected in any sample.

### Polymorphisms in *Pfdhfr* and *Pfdhps* genes

SP resistance markers were evaluated based on the combination of mutations in *pfdhfr* and *pfdhps.* Overall, 822/897 (91.6%) samples carried *pfdhfr* and *pfdhps* quintuple mutant (IRNGE; 94.1% in 2021 and 90.7% in 2022, *p* = 0.103; Table 2). Comparisons by region indicated a higher prevalence of quintuple mutants in the South of the country (174/182 [97%]) as compared to Centre (237/270 [88%]) and North (186/206 [90%]; *p* = 0.018) in 2022, which was independent of age group (older/younger than 5 years), gender and parasite density category (below or above 500 parasites/µL; **Table 2** and **Supplementary Table 4**). No statistically significant differences were observed in the prevalence of quintuple mutants by age groups, gender or parasite density (**Supplementary Tables 3 and 4**). Eleven samples carried *pfdhps* variant A581G (five in 2021 [1.6%] and six in 2022 [0.8%]). Ten of the A581G samples were detected in the North or Centre of the country and one in the South (**Table 2**; detail by province in **Supplementary Tables 1 and 2**). All A581G mutants with available data at other *dhps* loci (*n* = 10) were accompanied by K540E mutation.

Following previous reports from 2018^19^, we looked for variation at codon 436 of *pfdhps*. Four variants were identified (A/C/F/H), with alleles A and F as the most common substitutions (93% of mutants in 2021 and 92% in 2022). South to North prevalence of *pfdhps* S436ACFH ranged from 1.4 to 23.7% in 2021 and from 2.1 to 24.4% in 2022 (**Table 2** and **Supplementary Tables 3 and 4**). Prevalence of *pfdhps* 436 mutants was higher in children below 5 years of age (71/485 [15%]) compared to older children (7/309 [2%]; odds ratio for > 5 years in multivariate analysis = 0.24 (95% CI 0.1, 0.6); *p* < 0.001) (**Supplementary Table 4**). No mutations at *pfdhps* codon 431 were detected.

### Polymorphisms in *Pfcrt* and *Pfmdr1*

All but one sample were wild type at codons 72–76 of *pfcrt* (CVMNK haplotype). A single CVIET haplotype sample was detected in Nampula in 2022 (**Supplementary Table 2**). *pfmdr1* mutation Y184F prevalence also presented a geographical South-North gradient, being lowest in the South (< 60%) and higher in the North of the country both in 2021 and 2022 (> 76.3%; *p* = 0.001; Table 2) independently of age, gender or parasite density (**Supplementary Tables 3 and 4**). Mutations in codons *pfmdr1*-86 and *pfmdr1*-1246 were rare (< 1%; **Supplementary Tables 1 and 2**), and no variations were detected at *pfmdr1*-1034 and *pfmdr1*-1042 positions in any sample.

**Table 2:** Prevalence of antimalarial resistance markers during 2021 and 2022 rainy seasons in Mozambique.

2021	All regions	South	Centre	North	*p*-value
*pfk13*					
Validated mutants	0/311 (0%)	0/72 (0%)	0/108 (0%)	0/131 (0%)	-
Any nsyn mutants*	6/297 (2%)	0 (0%)	2/102 (2%)	4/125 (3.2%)	0.439
*pfdhfr*					
N51I	312/312 (100%)	72/72 (100%)	107/107 (100%)	133/133 (100%)	-
C59R	311/312 (99.7%)	72/72 (100%)	106/107 (99.1%)	133/133 (100%)	0.383
S108N	313/313 (100%)	72/72 (100%)	107/107 (100%)	134/134 (100%)	-
triple 51-59-108	238/239 (99.6%)	70/70 (100%)	75/76 (98.7%)	93/93 (100%)	0.341
*pfdhps*					
S436A/C/F/H	41/313 (13.1%)	1/71 (1.4%)	8/106 (7.6%)	32/135 (23.7%)	< 0.001
A437G	298/312 (95.5%)	69/71 (97.2%)	101/106 (95.3%)	128/135 (94.8%)	0.730
K540E	296/313 (94.6%)	70/72 (97.2%)	101/107 (94.4%)	125/134 (93.3%)	0.491
A581G	5/317 (1.6%)	0/72 (0%)	3/110 (2.7%)	2/135 (1.5%)	0.350
double 437–540	225/239 (94.1%)	68/70 (97.14%)	71/76 (93.4%)	86/93 (92.5%)	0.431
*pfdhfr/pfdhps* quintuple	225/239 (94.1%)	68/70 (97.1%)	71/76 (93.4%)	86/93 (92.5%)	0.431
*pfcrt* 72–76 CVIET	0/317 (0%)	0/72 (0%)	0/109 (0%)	0/136 (0%)	-
*pfmdr1* Y184F	216/313 (69%)	37/71 (52.1%)	76/107 (71%)	103/135 (76.3%)	0.001
**2022**	**All regions**	**South**	**Centre**	**North**	**p-value**
*pfk13*					
Validated mutants	0/794 (0%)	0/188 (0%)	0/339 (0%)	0/267 (0%)	-
Any nsyn mutants*	14/772 (1.8%)	2/184 (1.1%)	3/328 (0.9%)	8/260 (3.1%)	0.073
*pfdhfr*					
N51I	795/802 (99.1%)	189/190 (99.5%)	336/341 (98.5%)	270/271 (99.6%)	0.294
C59R	796/802 (99.3%)	190/190 (100%)	335/341 (98.2%)	271/271 (100%)	0.017
S108N	804/805 (99.9%)	190/190 (100%)	342/343 (99.7%)	272/272 (100%)	0.510
triple 51-59-108	645/658 (98.0%)	181/182 (99.5%)	259/270 (95.9%)	205/206 (99.5%)	0.005
*pfdhps*					
S436A/C/F/H	78/803 (9.7%)	4/189 (2.1%)	8/343 (2.3%)	66/271 (24.4%)	< 0.001
A437G	757/803 (94.3%)	183/189 (96.8%)	321/343 (93.6%)	253/271 (93.4%)	0.233
K540E	751/801 (93.8%)	181/189 (95.8%)	319/341 (93.6%)	251/271 (92.6%)	0.381
A581G	6/801 (0.8%)	1/189 (0.5%)	3/341 (0.9%)	2/271 (0.7%)	0.904
double 437–540	609/658 (92.6%)	175/182 (96.2%)	247/270 (91.5%)	187/206 (90.8%)	0.090
*pfdhfr/pfdhps* quintuple	597/658 (90.7%)	174/182 (96.6%)	237/270 (87.8%)	186/206 (90.3%)	0.018
*pfcrt* 72–76 CVIET	1/804 (0.1%)	0/189 (0%)	0/342 (0%)	1/273 (0.4%)	0.378
*pfmdr1* Y184F	565/803 (70.4%)	114/190 (60%)	242/343 (70.6%)	209/270 (77.4%)	< 0.001

*nsyn: non-synonymous mutation

**Table 3:** *pfk13* non-synonymous mutations detected in samples from 2021 and 2022 rainy seasons in Mozambique.

	2021				2022			
	**All regions** (297)	**South** (68)	**Centre** (102)	**North** (125)	**All regions** (772)	**South** (184)	**Centre** (328)	**North** (260)
G436S					**1**			1
G449S					**1**	1		
A481D					**1**		1	
C532S	**1**			1	**1**			1
G548D					**1**			1
A557V	**1**		1					
R561C					**1**			1
S576L					**1**	1		
A578S	**1**			1	**2**			2
A582V					**1**		1	
E602D	**2**			2				
Q613E	**1**		1		**1**			1
Y630F					**1**			1
L462S, M476T					**1**		1	
N554S, G453S					**1**			
Total	6	0	2	4	14	2	3	8

## Genetic diversity and population structure in 2022 rainy season

**Fig. 2:**
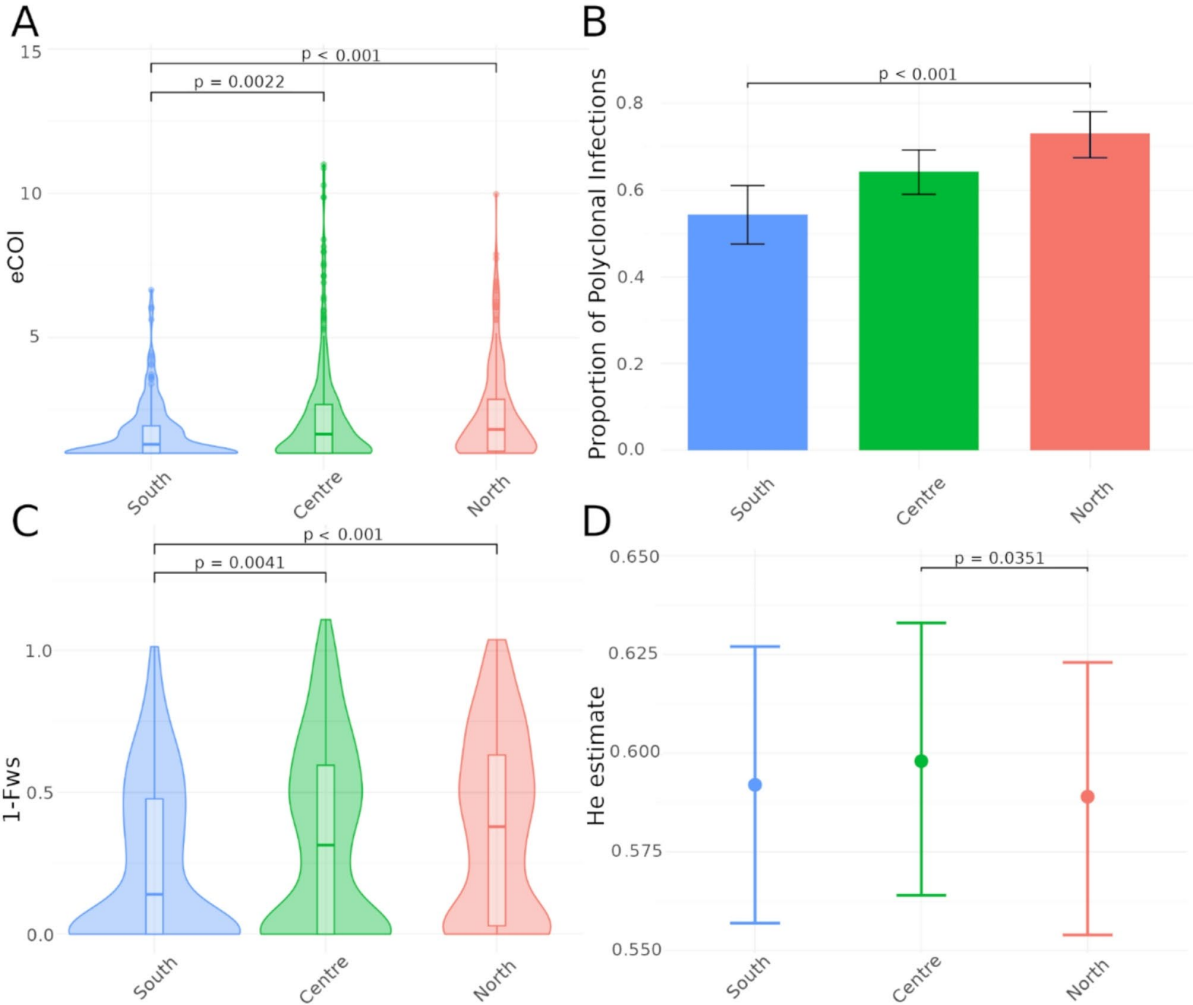
Genetic diversity of *P. falciparum* in samples from 2022 rainy season. **A**) effective COI; **B**) proportion of polyclonal infections; **C**) 1-Fws; **D**) heterozygosity estimate and 95% confidence intervals.

Genetic diversity was estimated using data from 165 microhaplotypes. The analysis focused on 2022 samples due to its wider geographical coverage and larger sample size per region (**Supplementary Fig. 2**). Median naive COI of all 2022 infections was 2 (IQR 1, 4). Median eCOI, which incorporates intra-host relatedness between clones (see Methods), was 1.6 (IQR 1, 2.6). eCOI was higher in the North (1.8, IQR 1.1, 2.9) and Centre (1.7, IQR 1, 2.7) as compared to the South (1.3, IQR 1, 1.9; *p* < 0.05; Fig. 2A) Similarly, the proportion of polyclonal infections was highest for the North (193/264, 73%), followed by the Centre (216/336, 64%) and lowest in the South (112/206, 54%; *p* < 0.001; Fig. 2B). Intra-individual heterozygosity measured by 1-Fws showed a similar north-south gradient, suggesting inbreeding in the parasite population in southern provinces (Fig. 2C). Statistically significant differences in eCOI, proportion of polyclonal infections and 1-Fws were maintained in the North and Center as compared to South after correcting for the confounding effect of age, gender or parasite density in multivariate regression models (**Supplementary Table 5)**. The intra-host diversity metrics results stratified at province level is provided in the Supplementary material (**Supplementary Fig. 3**). Population mean expected heterozygosity (H_E_) at 165 microhaplotype loci was 0.54 (95% CI 0.53, 0.55), ranging from < 0.01 to 0.92. Comparisons between the three areas indicated highest H_E_ in Centre provinces (Fig. 2D). The exploratory analysis of genetic diversity metrics for 2021 (*n* = 321) showed no differences in intra-individual metrics as compared to 2022, but lower H_E_, which can be attributable to low sample size and lower number of provinces included within each region (**Supplementary Fig. 4**).

**Fig. 3:**
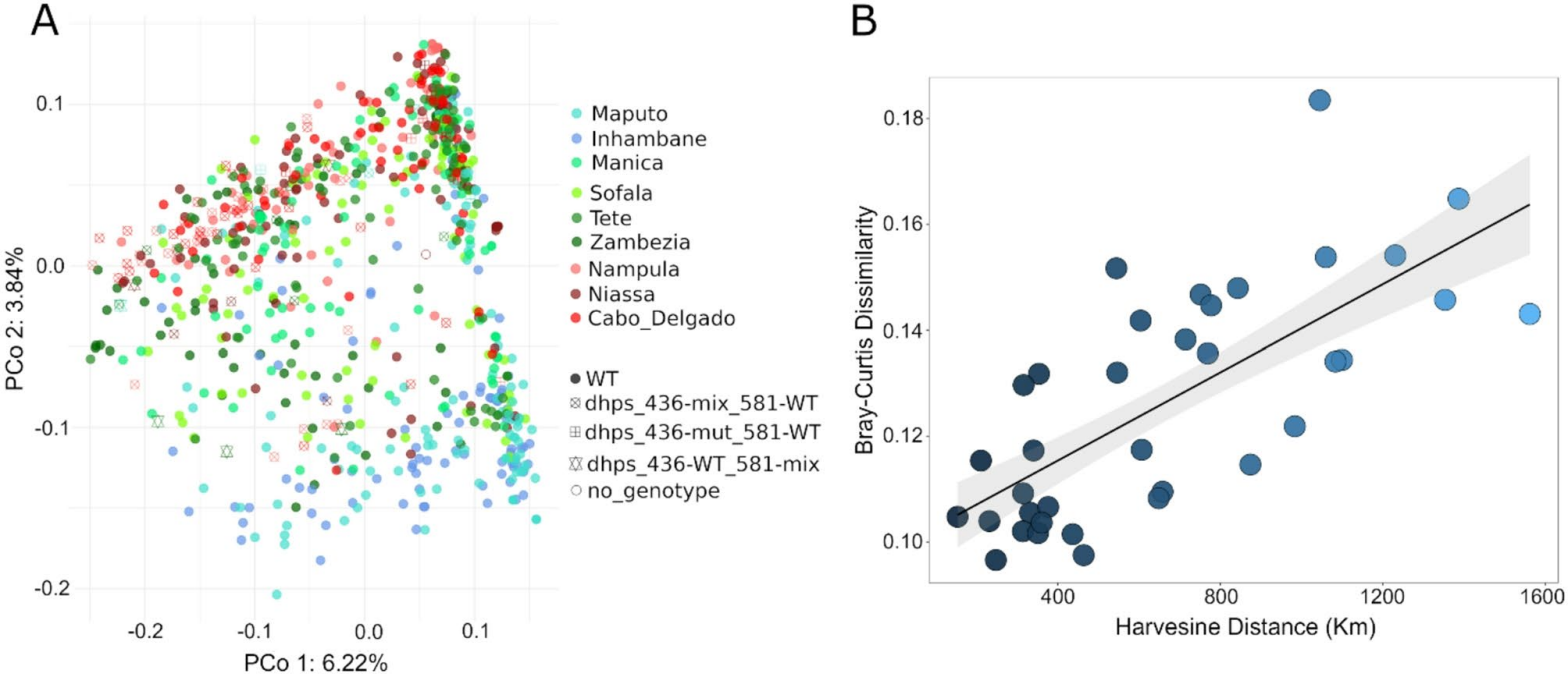
Population structure in samples from 2022 rainy season. **A**) Principal Coordinates Analysis with allele frequencies. Colors indicate administrative regions (red tones, North; green tones, Centre; blue tones, South). Samples with mutations in *pfdhp-s*436 and *pfdhps*-581 codons are indicated with hollow shapes. Bray-Curtis was used as the dissimilarity metric. **B**) Permutational Mantel test of Bray-Curtis distance calculated from population allele frequencies vs. geographic distance (Mantel’s rho = 0.73 *p* = 0.001; Spearman’s test with 10000 permutations).

Population structure was determined by clustering analysis of Principal Coordinates (PCoA). PCoA coordinates 1 and 2 allowed to differentiate South from North populations, whereas samples from central provinces showed more proximity to those in the North. As expected, based on prevalence data, most samples with *pfdhps*-436 clustered in the North, but there was no further particular sub-clustering of this group (Fig. 3A). Correlation of Bray-Curtis distance from population allele frequencies with the geographical distance between samples was significant (Mantel’s rho = 0.73 *p* = 0.001; Fig. 3B), further supporting geographical patterns based on latitude.

### Population structure of *Pfdhps* allelic haplotypes

**Fig. 4:**
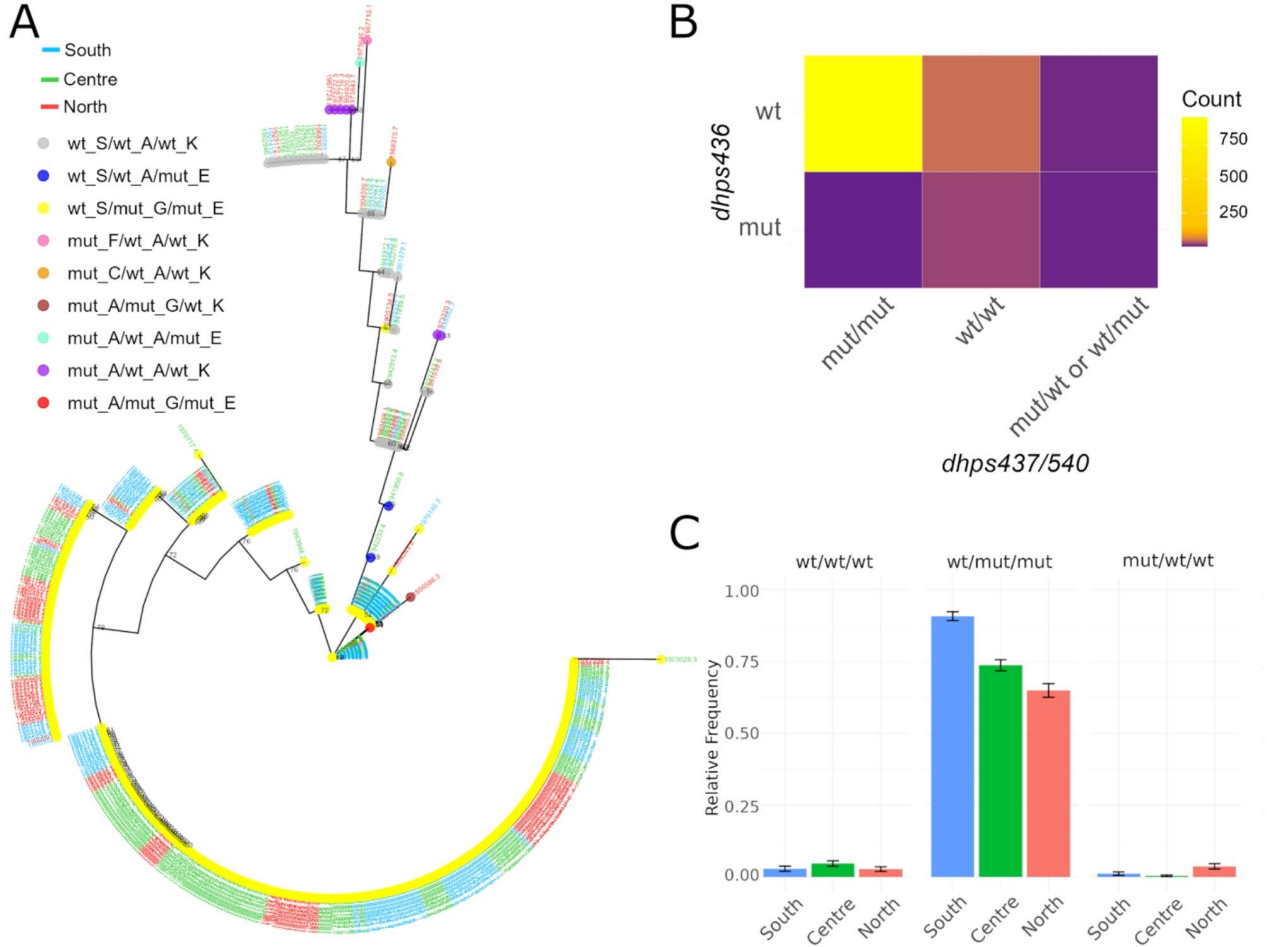
Population structure of *pfdhps*-436/437/540 haplotypes for 2022 rainy season in Mozambique. **A**) Maximum likelihood phylogeny. Branch support (> 50%) from 1000 bootstraps is shown. Tips are colored by genotype and labeled by region. **B**) Heatmap of genotype occurrences. **C**) Relative frequency of haplotypes for each region with error bars.

To further investigate the evolutionary history of *pfdhps* mutations, we analyzed 607 monoallelic infections for 6 loci covering or flanking the *pfdhps* gene (total concatenated sequences 1143 nucleotides). The set included 12 *pfdhps-*436 mutants: 11 from the North (9 S436A, 1 S436C, 1 S436F), and 1 from the South (S436A). Phylogenetic reconstruction focusing on *pfdhps-*436/437/540 haplotypes indicated a strong differentiation between the most common wild-type/mutant/mutant (wt/mut/mut) haplotype and the mut/wt/wt, with the latter more closely related to wt/wt/wt (Fig. 4A). Infections with a mutant *pfdhps-*436 were more likely to be wild type for *pfdhps-*437/540 (*p* < 0.001; Cramér’s V = 0.543; Fig. 4B), while wild type *pfdhps-*436 infections were more likely to be mutant for both *pfdhps-*437/540. Triple mutants for the *pfdhps-*437/540 codons were rare. Overall, haplotypes wt/wt/wt, wt/mut/mut, and mut/wt/wt were significantly associated with populations at regional level, although the association was weak (*p* < 0.001; Cramér’s V = 0.108) (Fig. 4). The distribution of all three haplotypes across regions was significantly different from what would be expected by chance (wt/wt/wt: *p* = 0.017, Cramér’s V = 0.063; wt/mut/mut: *p* < 0.001, Cramér’s V = 0.101; mut/wt/wt: *p* < 0.001, Cramér’s V = 0.082). At the province level, statistically significant associations were moderate for wt/mut/mut (*p* < 0.001, Cramér’s V = 0.26) and weak for mut/wt/wt (*p* < 0.001, Cramér’s V = 0.132; **Supplementary Fig. 5**).

## Effect of seasonality on drug resistance and genetic diversity

The provinces of Maputo and Manica were used to study the effect of interannual malaria seasonality on drug resistance markers in 2022. According to the DHIS2 data, the RDT positivity rate among less than 5 year old children (who sought care at either a heath facility or community healthcare worker level differed significantly in Manica province between rainy (January to May; median 59.5% [IQR 41.1–72.2]) and dry season (June to September; 47.8% [31.0-64.2]; *p* < 0.001), but was similar throughout the year in Maputo (7.2% [3.3–10.6] in rainy vs. 5.7% [3.0-8.1] in dry; *p* = 0.230; **Supplementary Fig. 6**). One-hundred samples were randomly selected per province, out of which 70 and 85 were confirmed qPCR positive and produced valid sequences for Maputo and Manica, respectively. No statistically significant differences in main demographic or parasitological characteristics were found between seasons (Tables 4). Prevalence of *pfmdr1*-Y184F in Maputo province was higher in the dry season, and both *pfmdr1*-Y184F and *pfk13* non-synonymous mutations were more common in the dry season when adjusting for age, gender and parasite levels (Table 4 and **Supplementary Table 6**). No differences were found in the percentage of polyclonal infections, eCOI and 1-Fws (in rainy compared to dry seasons (Table 4), neither after adjusting by age gender or parasite density. H_E_ was higher in rainy as compared to dry season in Manica (*p* = 0.017) but not in Maputo.

**Table 4:** Demographic characteristics, parasitemia and genetic markers of drug resistance and diversity in Maputo and Manica provinces, by transmission season in 2022.

	Maputo			Manica		
	Rainy	Dry	*p*-value	Rainy	Dry	*p*-value
**N**	100	70		81	85	-
**Patient characteristics**:						
Female, n (%)	49 (49%)	33 (47.1%)	0.763	37 (45.7%)	45 (52.9%)	0.350
Age, median yr (IQR)	15 (6, 28)	8 (4, 29)	0.118	6 (4, 8)	5 (4, 7)	0.738
Parasitemia, median parasites/µL (IQR)	12,041 (1389, 42501)	11,491 (1367, 33176)	0.568	8648 (799, 37875)	24,865 (1395, 61019)	0.056
**Drug resistance markers**,** n/N (%)**:						
*pfk13*						
Validated mutants	0/99 (0%)	1/68 (1.5%)	0.226	0/78 (0%)	0/76 (0%)	-
Any nsyn mutants*	2/95 (2.1%)	4/66 (6.1%)	0.193	1/76 (1.3%)	0/72 (0%)	0.329
*pfdhfr* triple 51–59-108	94/94 (100%)	62/62 (100%)	-	56/59 (94.9%)	60/64 (93.8%)	0.780
*pfdhps* double 437–540	88/94 (93.6%)	58/62 (93.6%)	0.986	52/59 (88.1%)	62/64 (96.9%)	0.063
*pfdhfr/pfdhps* quintuple	88/94 (93.6%)	58/62 (93.5%)	0.986	49/59 (83.1%)	58/64 (90.6%)	0.212
*pfcrt* 72–76 CVIET	0/100 (0%)	0/70 (0%)	-	0/78 (0%)	0/77 (0%)	-
*pfmdr1* Y184 F	54/100 (54.0%)	52/70 (74.3%)	0.007	52/79 (65.8%)	50/76 (65.8%)	0.997
**Diversity metrics**:						
eCOI, median (IQR)	1.1 (1, 1.9)	1.5 (1, 2.2)	0.161	1.8 (1, 2.2)	1.7 (1, 2.4)	0.614
% polyclonal, median (IQR)	50% (40.4, 59.6)	63.2% (51.4, 73.7)	0.114	51.5% (50.4, 71.6)	69.1% (57.4, 78.8)	0.386
1-Fws, median (IQR)	0.12 (0, 0.53)	0.23 (0, 0.57)	0.200	0.39 (0, 0.54)	0.30 (0, 0.57)	0.852
H_E_, median (IQR)	0.58 (0.55, 0.61)	0.58 (0.55, 0.60)	0.295	0.58 (0.54, 0.61)	0.56 (0.54, 0.59)	0.017

*nsyn: non synonymous mutations

## Discussion

In this study, we applied a sensitive, high-throughput genomic assay to detect signals of drug-resistance and genetic diversity in malaria parasites from nine provinces and three regions in Mozambique, representative of the different malaria transmission strata in the country. We found that (1) markers of partial resistance to artemisinin derivatives and resistance to piperaquine partner drug were residual or absent; (2) there was a high prevalence of *pfdhfr/pfdhps* quintuple mutants in all three regions, but the prevalence of sextuple mutants remains very low; (3) the mutations were not influenced by gender and parasite density; (4) there was a south-to-north gradient in genetic complexity and prevalence of mutations at *pfdhps-*436.

The continued monitoring and identification of drug-resistant *P. falciparum* parasites is crucial in the fight against malaria. Out results show the absence of validated or candidate mutations associated with partial artemisinin resistance in 2022, in line with previous studies conducted in the country with samples from before 2021^28-32^. However, 17 non-synonymous mutations were detected in the *pfk13* gene, two of which had already been reported in Mozambique (G436S^19^ and A578S^19,29^). To date no mutants detected in Mozambique have been associated with a resistant phenotype, although a recent case of imported malaria from Mozambique to Portugal reported elevated ex vivo half-maximal inhibitory concentrations values to dihydroartemisinin in the presence of *pfk13-*I416V mutation^33^. We did not find that mutation in this study. The absence of *pfpm2* mutants (and any other candidate markers of piperaquine resistance)^27^ is reassuring for an adequate efficacy of DP, currently being used as the ACT of choice in mass drug administration campaigns led by NMCP. However, the risk of emergence of ACT resistance in Mozambique remains due to continuous drug pressure and the recent emergence of artemisinin partial resistance in neighboring Tanzania^9,34^ and other East African countries^6,8,10,11^.

The overall rate of *pfdhfr-pfdhps* quintuple was high across the three regions and, despite South still had the highest rates, there was a notable increase in the North (90.3% in 2022) compared to levels observed in 2015 (40%) and 2018 (72%)^19^, leading to an homogenization of *pfdhfr-pfdhps* prevalences across the country. The fact that sextuple mutants were uncommon, together with evidence generated from clinical studies^35^, suggests that chemopreventive strategies based on the use of SP still retain some chemopreventive effect in the current parasite molecular resistance background^35^.

A clear North-South divide was also found in the distribution of *pfdhps*-S436A, *pfmdr1*-Y184F and in the genetic complexity of *P. falciparum*, with higher prevalence of mutations and diversity found in the high burden provinces of the North. PCoA and a Mantel test support a notable regional structuring of parasite populations in line with previous observations in years 2015 and 2018 derived from WGS data^19^. The large geographical distance between regions, local heterogeneity in intervention coverage and difficult inter-regional population movement, likely contribute to ecological barriers with restricted parasite migration and limited gene flow. Interestingly, mutations in *pfdhps*-S436A clustered with those wild-type at 437 and 540 codons, suggesting a common origin. In the case of *mdr1*-Y184F, not only levels in the North were relatively higher, but prevalence in the region increased from 50 to 56% in 2015/18 to 77% in 2022^19^. Drivers of differences in these resistant markers are unclear. There are no differences in treatment policy in the North compared to the South of the country, but coverage of antimalarial interventions may vary between regions due to the unequal distribution of resources and security issues, what could exert differential drug pressures. In addition, we cannot exclude that a pilot implementation of SMC with amodiaquine-SP ongoing in Nampula province since 2020 could contribute to expansion of *pfmdr1*-Y184F, as previously observed in other countries^36^.

Parasite genetic diversity has been proposed as an indicator to characterize the key drivers of ongoing transmission, to identify foci and to discriminate between indigenous and imported cases in areas approaching elimination^37^. It has been suggested that reductions in *P. falciparum* genetic diversity and increased similarity due to inbreeding and recent common ancestry reflect bottlenecks in parasite population driven by control and elimination efforts^37,38^. Our data indicates that intra-host metrics of genetic diversity align with a South-to-North increase in regional transmission intensity measured with traditional epidemiological indicators. Although the study was not designed to capture fine scale/local changes, these results add-up to the reports supporting the use of *P. falciparum* genetic diversity measures to supplement traditional surveillance for improving stratification, monitoring and impact evaluations in different epidemiological contexts, especially where epidemiologic surveillance data is sparse^39^. This use case still requires development of analytical and interpretative tools to infer malaria burden and effectiveness of interventions, as well as validation of sampling frameworks^38,40^.

One of the concerns in the design of sampling schemes for malaria molecular surveillance is the time of collection, especially in areas where malaria transmission is highly seasonal^23^. We addressed this question by sequencing samples from the dry season in Manica (a province with marked seasonality in malaria positivity rates) and Maputo (a province with no seasonality, selected as control). Differences in both *pfk13* and *pfmdr1* Y184F were observed in the Maputo control province when adjusting for demographic factors, whereas no impact was seen in Manica (although comparisons were limited by sample size and lack of variation). Overall data suggests that time of sampling may have some impact in the genetic metrics measured especially in dry season for low transmission areas, where outbreaks, cluster effects or importation events, among other situations, could impact in both increasing or decreasing point prevalence estimates.

The study presents some limitations. First, different sampling schemes were followed in 2021 and 2022. Surveys in 2021 were conducted at multiple randomized health facilities but in a limited number of provinces, some of which had very low sample sizes. Surveys in 2022 covered a wider, more balanced geographical area, but health facilities were selected by convenience. Although most results showed consistency across years, we cannot exclude biases due to this heterogeneity. Second, we acknowledge some technical limitations that should be considered in data interpretation, such as the exclusion of polyclonal infections in haplotype reconstruction, or low densities samples in the estimation of *pfpm2* CNV. Finally, although genetic markers of resistance provide a baseline and early warning signal for antimalarial resistance, drug efficacy should ultimately be confirmed using therapeutic and/or chemoprevention efficacy studies.

## Conclusions

*P. falciparum* parasites circulating in Mozambique in 2021 and 2022 were characterized by the absence of molecular markers of partial resistance to artemisinin and of resistance to ACT partner drugs, suggesting an appropriate efficacy of artemisinin-based combination therapy for *P. falciparum* treatment. Infections presented high rates of *pfdhfr*/*pfdhps* quintuple haplotype, which increased in the North compared to data from 2018, but were mostly wild type for *dhps*-581 mutation that would confer additional SP resistance. Parasite populations in Northern provinces also present higher genetic diversity and a cluster of *pfdhps*-436 mutants, pointing towards the importance of continuous surveillance of these mutations in Mozambique. The results of this study are informative for public health decision makers on discussions about malaria treatment and control.

## Methods

### Study design and sample collection

In 2021, malaria patients aged 2–10 years old were recruited as part of the National Health Facility Survey (NMCP, Ministry of Health) that evaluated quality of malaria case management at public outpatient clinics in six provinces of Mozambique (Maputo, Inhambane, Manica, Zambézia, Niassa and Nampula). 40 health facilities were randomized out of all eligible centers to maximize representativity (Fig. 1). In 2022, targeted sampling was conducted at selected health facilities in Maputo province (all ages > 6 months) and Inhambane, Manica, Zambézia, Sofala and Manica (children 2–10 years old). Samples from Nampula were obtained from children aged 3 months to 5 years old at health facilities in areas where seasonal malaria chemoprevention was implemented. Samples from Tete and Cabo Delgado were obtained from the 2022 therapeutic efficacy survey (children aged 6 months to 5 years old), with an additional inclusion criterion of parasite density > 2500 parasites per microliter by microscopy^20^. Additional surveys were conducted in 2022 during dry season (June to October) for Maputo and Manica provinces to study the effect of malaria seasonality on molecular markers of antimalarial resistance and parasite diversity.

In all surveys, patients presenting with a confirmed diagnosis of uncomplicated *P. falciparum* malaria by RDT were invited to participate^20^. After they provided informed consent, two to four 50 µL DBS were prepared onto one or two filter papers through finger prick. DBS were identified with anonymous barcodes, air-dried during 72 h and stored at 4 °C in sealed bags with silica gel until laboratory processing.

### Genomic DNA extraction and quantification

Genomic DNA was extracted from DBS samples using a Tween-Chelex based protocol as described by Brokhattingen et al.^30^, with some modifications. Five mm (~ 12,5 µL blood) discs were cut from each DBS into 96-well deep well plates with a manual puncher. One mL of freshly made 0.5% Tween 20^®^ detergent diluted in PBS was added to each well plate containing a DBS punch and incubated overnight in a thermomixer at 15ºC and 300 rpm. The next morning, the supernatant was removed, 1 mL of fresh PBS was added per well and the plate was briefly vortexed and then incubated at 4ºC for 30 min. After incubation, the liquid was aspirated, and 150 µL of a solution of 10% Chelex (C7901, Merck) in molecular grade water was added. The samples were incubated at 95ºC in a water bath for 15 min with gentle vortexing every 5 min. The plate was then centrifuged for 5 min at 1500 rpm to pellet the Chelex^®^ beads. Supernatant (approximately 130 µL) containing the eluted DNA was transferred to a new PCR 96 well plate, centrifuged again and finally 100 uL transferred to barcoded tubes placed on 96 well plate (Wilmut).

*P. falciparum* infection was confirmed in all DNA samples by qPCR targeting the 18 S rRNA gene on an ABI PRISM 7500 HT Real-Time System (Applied Biosystems), as previously described^41^. Parasite density was quantified by extrapolation to an external standard curve composed of six 1:10 dilutions of 3D7 (MRA-151, MR4, Bei Resources) cultured parasites in whole blood spotted onto filter paper (range 100.000 to 1 parasites/µl). DNA was stored at -20ºC until sequencing.

### Amplicon-based sequencing

Sequencing was performed using the MAD^4^HatTeR multiplex amplicon sequencing panel using CleanPlex reagents and CleanMag Magnetic Beads (Paragon Genomic Inc, California, USA) as previously described^22^. Briefly, we used two multiplexed PCR reactions with primer pools D1.1, R1.2 (reaction 1) and R2.1 (reaction2). Together, these primer pools target 241 *P. falciparum* loci of 225–300 bp^22^. Multiplex PCR was performed with 10 cycles if parasite density ≥ 500 parasites/µL, or 20 cycles for those samples with < 500 parasites/µL. Following multiplex PCR, reactions proceeded in a single tube for each sample, where products were bead-cleaned, digested, and indexed via PCR to generate Illumina-compatible libraries. A randomly selected subset of 10 libraries from each full plate was assessed using automated capillary electrophoresis in a TapeStation 4150 (Agilent technologies, California, USA) to confirm library quality (size and concentration). Finally, libraries from each sample were pooled adjusting volumes based on parasitemia, and the pool was bead-cleaned using a 1X bead ratio to remove primer dimers. Pooled libraries were run on an agarose gel, from which the amplicon-sized band was excised. DNA was extracted using Monarch^®^ DNA Gel Extraction Kit (New England Biolabs Inc., Massachusetts, USA), and products were quantified using a TapeStation and a Qubit fluorometer. For 2021 samples, pools contained 96 samples and were 150 paired-end sequenced in a MiSeq System with v2-300 cycles reagents (Illumina, USA) at CISM laboratory; for 2022 samples, pools of 288 samples were also sequenced with 150 paired-end reads in a NextSeq 2000 System using P1 reagents at ISGlobal laboratory.

Positive (*n* = 2, matching the parasitemia category of the samples in the plate) and negative (*n* = 2) controls were included in every library preparation plate to control for run quality and contaminations. Positive controls were prepared from *P. falciparum* laboratory strains 3D7 (MRA-151), HB3 (MRA-155), Dd2 (MRA-156 and MRA-1255). Cultures were synchronized in the ring stage, mixed with uninfected human whole blood to obtain a range of parasite densities (1, 10, 100, 1,000, 10,000 and 100.000 parasites/µL) and spotted onto filter paper. Negative controls were prepared from *P. falciparum* negative DBS.

### Sequence data analysis

FASTQ files were subjected to filtering, demultiplexing and allele inference using a Nextflow-based pipeline version 0.1.8 (https://github.com/EPPIcenter/mad4hatter)^22^. The 3D7 genome sequence was used as reference for alternative allele calling (https://github.com/EPPIcenter/mad4hatter/blob/main/resources/v4/ALL_refseq.fa). The resulting allele tables were subsequently filtered based on read counts and coverage across loci within a sample and across samples. Alleles with fewer reads than the maximum observed reads in any locus for negative controls were removed, along with alleles with < 1% within-sample frequency.

Prevalence of antimalarial drug resistance markers was defined as the number of *P. falciparum* infections carrying mutant alleles (including mixed infections) out of total infections with valid allele calls per each locus. Reconstruction of *pfdhps* double, *pfdhfr* triple and *pfdhfr*/*pfdhps* quintuple haplotypes was done for samples with no mixed genotypes at selected loci and for those with allele frequency > 95%. Within-host and population level metrics of diversity were calculated from diversity locus (*n* = 165) as recently described^30^. Samples with < 50 diversity loci covered at a read depth of at least 100, and diversity loci with < 100 samples covering them with at least 100 reads were filtered out. Intra-host complexity of infection (COI) and effective COI (eCOI) were estimated from polyallelic genomics data using a Markov Chain Monte Carlo (MCMC)-based approach implemented in MOIRE v3.4.0 (https://github.com/EPPIcenter/moire). eCOI considers within-host relatedness, and can be interpreted as the expected COI if population diversity was infinite (heterozygosity = 1). Polyclonal infections were defined as having eCOI > 1.1^30^. Wright’s inbreeding co-efficient (Fws) was calculated as 1-Fws across all diversity loci as the allele heterozygosity of the individual (H_W_) relative to the population^42^. Genetic diversity of the parasite population was measured using expected heterozygosity (H_E_), i.e., the probability that two randomly selected parasites carry distinct alleles at each diversity locus^30^. Principal Coordinates Analyses (PCoA) of in-sample allele frequencies and presence/absence of alleles were done to visualize the differences between provinces and regions. Bray-Curtis dissimilarity matrices were computed using the R package vegan (https://vegandevs.github.io/vegan/)^43^. A permutational Mantel test (10000 permutations) utilizing Spearman’s correlation method was conducted to evaluate the correlation between genetic (Bray-Curtis dissimilarity) and geographic distances using vegan. Geographic coordinates for each province were used to calculate a Haversine matrix.

To infer the evolutionary history of the mutant alleles in *pfdhps*, we focused on six specific amplicons surrounding the *pfdhp*s gene covering positions 549,583 to 596,266 on chromosome 8 (549583–549807, 549960–550215, 550057–550318, 585331–585590, 585703–585949, and 585993–586266). Each amplicon was aligned separately with the R package msa^44^ and concatenated into a 1143-nucleotide sequence. Pairwise distances between infections were calculated, and an initial unrooted tree was constructed using the minimum evolution algorithm^45^. The best evolutionary model was identified using the Bayesian Information Criterion (BIC), and 1000 bootstrap replicates were performed for statistical support. Phylogenetic reconstruction was conducted with the Phangorn package in R^46^. Chi-squared tests assessed associations between *pfdhp*s-436 codon (mutant or wild type) and *pfdhp*s-437/540 codons, between haplotypes (wt/wt/wt, wt/mut/mut, and mut/wt/wt) and populations (region or province) and between frequency of each haplotype across regions and provinces. Cramér’s V was used to measure effect sizes. Haplotypes mix/wt/wt and mix/mix/wt were considered mut/wt/wt, and the rest of mixed haplotypes were excluded from the analysis. Single mutants for dhps437/540 were grouped into one category for the codon association analysis. The population association analysis only included haplotypes wt/wt/wt, wt/mut/mut, and mut/wt/wt.

### Copy number of *pfpm2*

MAD^2^HatTeR data was screened for CNV at *pfpm2* locus using read counts. A generalized additive model was constructed based on amplicon read counts for each sequencing run under the assumption that most amplicons correspond to single-copy genes. Fold change values were calculated from the difference between observed and expected read counts, and further normalized using the resulting fold change of single-copy laboratory 3D7 controls sequenced in each run to account for technical variation. Additionally, the 5% of amplicons with the highest read count variability across sequencing runs were excluded from the fold change calculation to reduce noise. Samples with normalized fold changes greater than 1.5 were flagged as potential CNV. The analysis was restricted to samples with a parasite density > 1000 parasites/µL, as lower parasitaemia samples are more prone to produce inconsistent read counts across runs^22^.

Samples with read fold-change > 1.5 were tested for *pfpm2* CNV adapting a previously published qPCR protocol^15^ by using *ubiquitin conjugated enzyme* (*pfuce*) as reference gene^47^. Reactions were set-up in an ABIPrism 7500 Thermocycler with Power SYBR Green master mix (ThermoFisher). Amplification efficiencies calculated from standard curve build with 3D7 genomic DNA were 0.90% for *pfpm2* and 0.81% for *pfuce*. Copy numbers were calculated using ddCt method and 3D7 (one *pfpm2* copy) as calibrator sample. Samples with CNV > 1.5 by qPCR were considered *pfpm2* duplications. DBS from a field isolate with confirmed four *pfpm2* copies were included as positive control (kindly donated by Prof. Didier Ménard, Institute Pasteur).

### Definitions and statistical analysis

Regions for geographical analysis were South (Maputo, Inhambane), Centre (Manica, Sofala, Zambézia, Tete) and North (Nampula, Niassa and Cabo Delgado). Maputo Province and Maputo City, which are administratively independent provinces, were considered as a single unit for sampling purposes and in secondary analysis at the province level. Maps were created using Python 3.9 from the public OpenStreetMap data.

The prevalence of infections carrying parasites with markers of antimalarial resistance were estimated per region, year and transmission season. Validated and associated markers of antimalarial drug resistance were considered as those included in WHO’s 2020 review report on antimalarial drug-resistance^13^. Rainy season was defined as the period between January 1st and May 30th, and samples collected after this date and up to October 31st were considered dry season. Malaria case data per each province and season was extracted from the Health Information System for Monitoring and Evaluation (SIS-MA, Ministry of Health, Mozambique). Health facilities reporting a minimum of 96 malaria rapid diagnostic tests per study month were included to avoid biases for health facility with sporadic testing patters (95% confidence interval and 80% power). Positivity rate was defined as the percentage of positive malaria RDTs out of the total tests conducted in each of the health facilities.

Statistical analyses were performed in Stata version 15.0 or R version 4.3.1. Chi-square test, Fisher’s exact were used to compare frequencies of resistance markers and differences in percentage of polyclonal infections by location and/or year. Kruskal–Wallis rank sum test was used for the comparison of the distribution of parasite densities, read counts, eMOI or 1-Fws between populations. Multivariate regression models for genetic metrics were build adjusting for the age (older/younger than 5 years), gender and parasite density (below or above 500 parasites/µL. Genome-wide He estimate and 95% confidence intervals were calculated by a linear mixed model fitting locus as a random effect. To compare the malaria positivity rates between the rainy and dry seasons within each province, the Wilcoxon rank sum test was employed. P-values lower than 0.05 was considered to indicate statistical significance.

### Ethical considerations

Clinical-demographic data and blood samples were collected only after written informed consent was obtained from participants and/or their accompanying adults. All RDT positive individuals were treated according to national treatment guidelines. Study protocols were approved by the Mozambican National Committee for Bioethics in Health (CNBS, references number 354/CNBS/2021 and 604/CNBS/21) and Hospital Clinic de Barcelona Ethics Review Committee (ref. HCB/2022/0097).

## Electronic supplementary material

Below is the link to the electronic supplementary material.

Supplementary Material 1

## Data Availability

Sequencing data is available at NCBI Sequence Read Archive (SRA) under BioProject PRJNA1107381 (https://www.ncbi.nlm.nih.gov/bioproject/PRJNA1107381): accession numbers SAMN41182041-SAMN41182361 (2021 samples), and SAMN41181143-SAMN41182039 and SAMN41180171-SAMN41180265 (2022 samples). The raw epidemiological data includes personal information and is protected by data privacy laws. Anonymized databases can be made available for research purposes from the corresponding author upon reasonable request by email. Requests for data will be reviewed in a three-month timeframe by Manhiça Health Research Center to verify that data sharing is not subject to any intellectual property or confidentiality obligations. If data can be shared, it will be released via a Data Transfer Agreement.
