# Supplementary material for: Antimalarial drug resistance and population structure of Plasmodium falciparum in Mozambique using genomic surveillance at health facilities in 2021 and 2022
Source: Sci Rep. 2025 Aug 11;15:29335. doi: 10.1038/s41598-025-02166-w (PMC12340125; doi:10.1038/s41598-025-02166-w)
Supplement: Supplementary file 1 — Supplementary Material 1 [file 41598_2025_2166_MOESM1_ESM.docx]

**SUPPLEMENTARY INFORMATION**

**Boene *et al.* Antimalarial drug resistance and population structure of *Plasmodium falciparum* in Mozambique using genomic surveillance at health facilities (2021-2022)**

**Supplementary Figures**

Supplementary Figure 1. Read depth for 24 amplicons covering drug resistance markers of interest

Supplementary Figure 2. Allele accumulation curves for 2021 and 2022 rainy seasons, by region.

Supplementary Figure 3. Genetic diversity of *P. falciparum* in samples from 2022 rainy season, by province.

Supplementary Figure 4. Genetic diversity in 2021 as compared to 2022 rainy season, by region.

Supplementary Figure 5. Relative frequency of *pfdhps*-436-437-540 haplotypes, by province.

Supplementary Figure 6. Malaria positivity rates for children under 5 year in Maputo and Manica provinces, by transmission season in 2022.

**Supplementary Tables**

Supplementary Table 1. Prevalence of antimalarial drug-resistance markers in 2021 rainy season, by province.

Supplementary Table 2. Prevalence of antimalarial drug-resistance markers in 2022, by province.

Supplementary Table 3. Multivariable logistic regression model for drug-resistance markers in 2021.

Supplementary Table 4. Multivariable logistic regression model for drug-reisstance markers in 2022.

Supplementary Table 5. Multivariable logistic regression model for factors associated with genetic diversity metrics in 2022.

Supplementary Table 6. Multivariable logistic regression model for factors associated with drug-resistance markers in the provinces of Maputo and Manica in 2022, including seasonality.

**Supplementary Figure 1. Read depth for 24 amplicons covering drug resistance markers of interest.**Reads per amplicon and sample are stratified by experiment runs conducted in 2021 (A, MiSeq system, Illumina; CISM) or 2022 samples (B, NextSeq system, Illumina; ISGlobal). Amplicon are labelled based on gene targeted with main codon of interest in brackets. White lines indicate median and interquartile range.

**
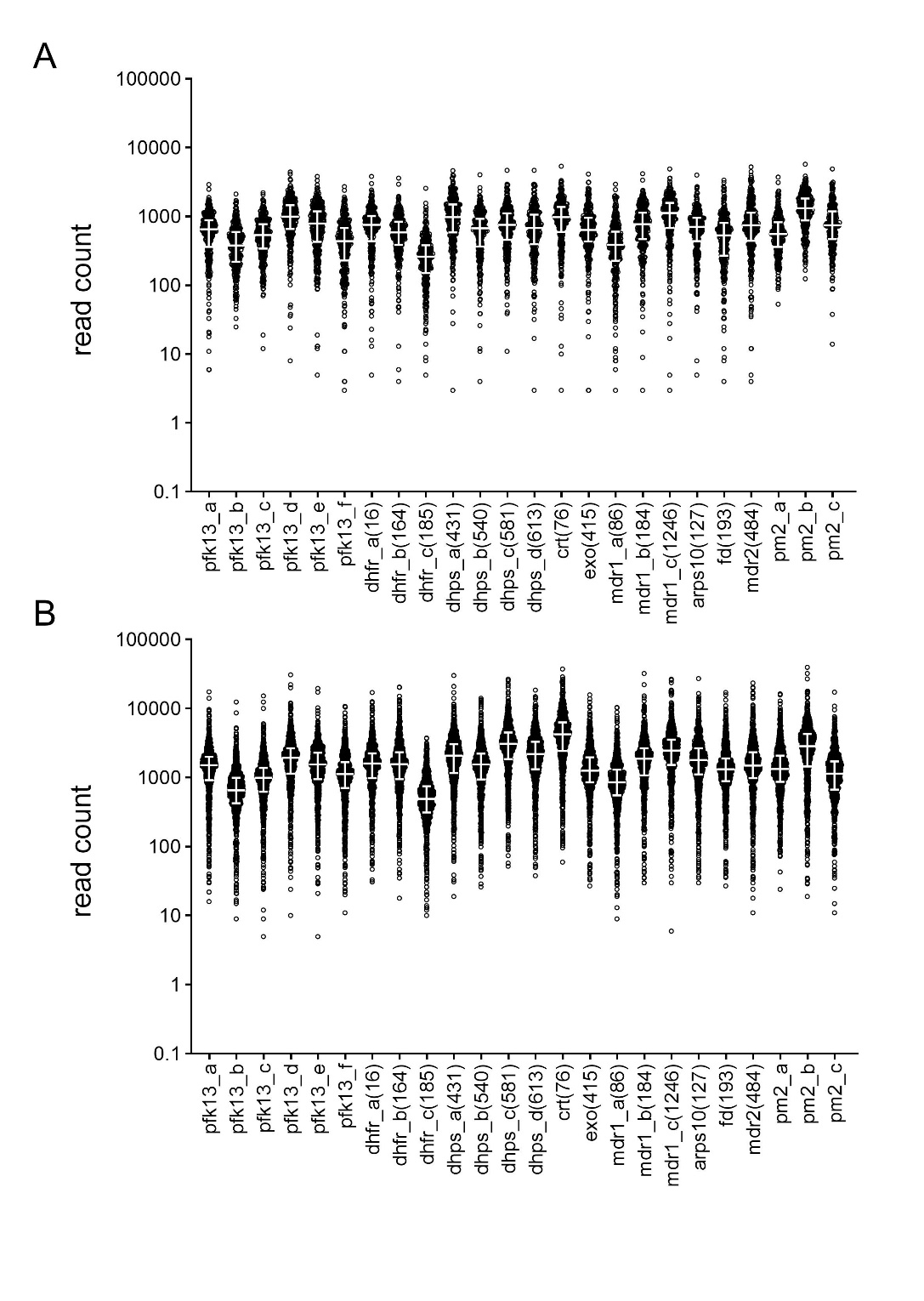
**

**Supplementary Figure 2. Allele accumulation curves for 2021 and 2022 rainy seasons, by region.** Number of new alleles accumulated at the 165 diversity amplicons with increasing sample size (x axis), using regional stratification in 2021 (A) and 2022 (B)

**
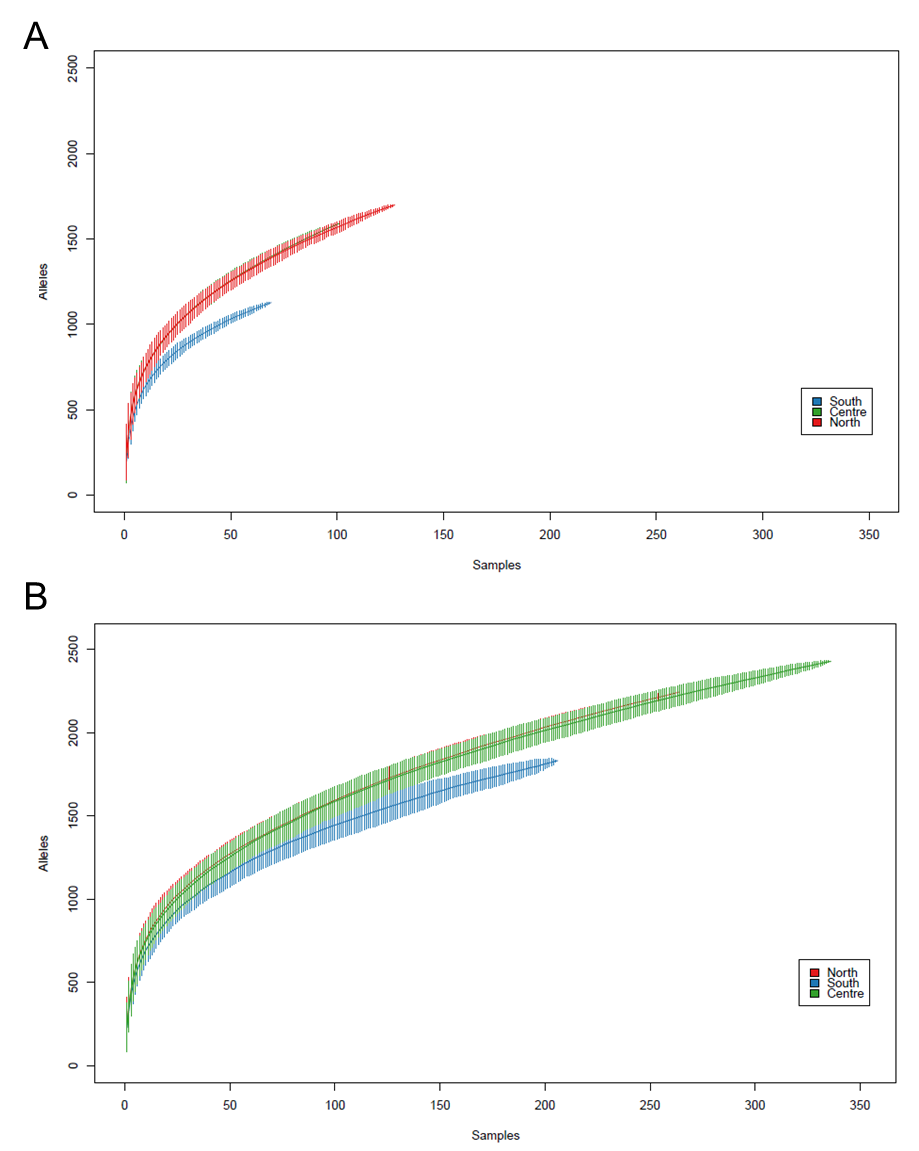
**

**Supplementary Figure 3. Genetic diversity of *P. falciparum* in samples from 2022 rainy season, by province.**  A) Effective COI; B) % of polyclonal infections; C) 1-Fws; D) heterozygosity estimate and 95% confidence intervals. P-values: ***< 0.001, **< 0.01, *< 0.05. Colors indicate administrative regions (red tones, North; green tones, Centre; blue tones, South)


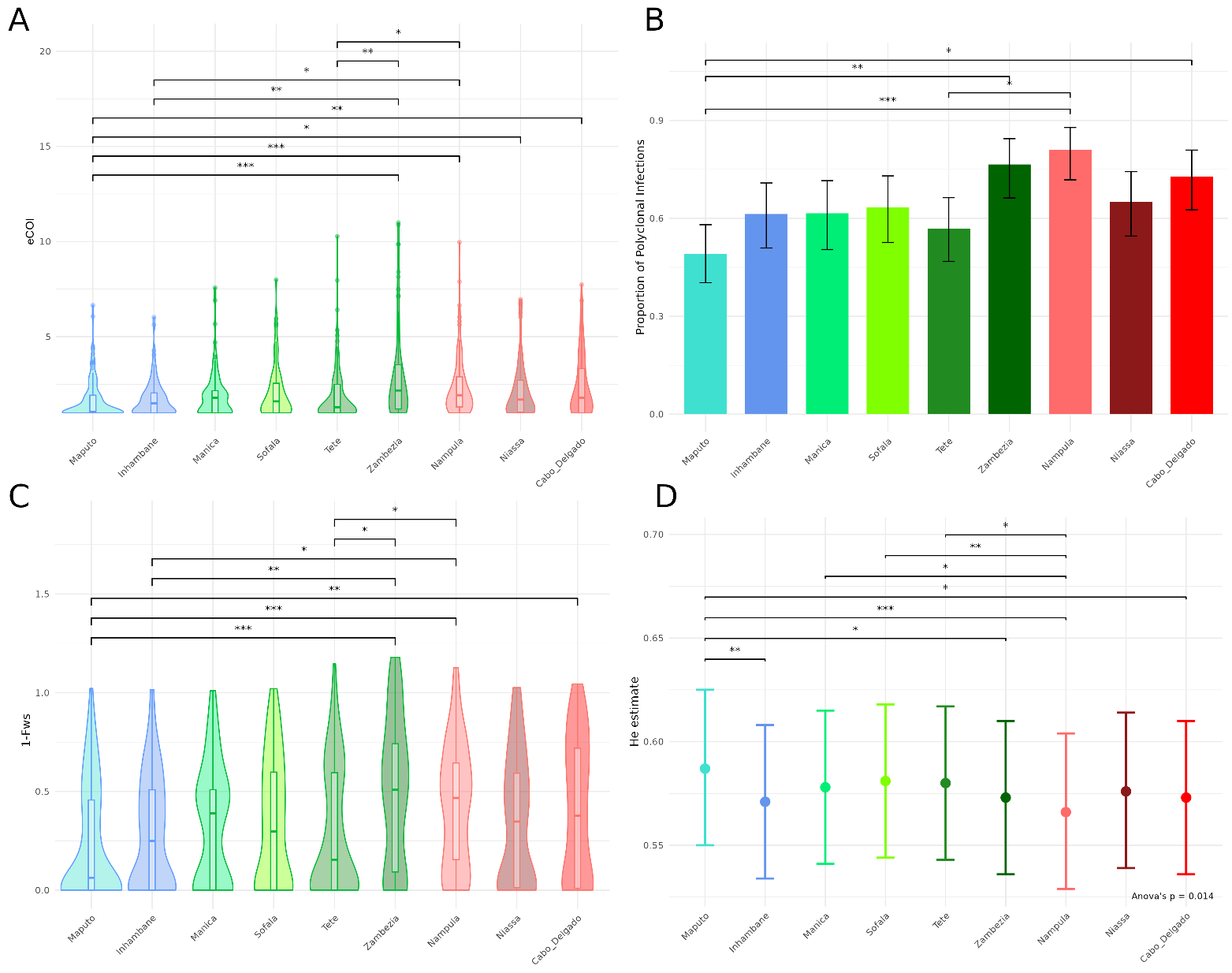


**Supplementary Figure 4. Genetic diversity in 2021 as compared to 2022 rainy season, by region.** A) effective COI; B) % of polyclonal infections; C) 1-Fws; D) heterozygosity and 95% confidence intervals

**
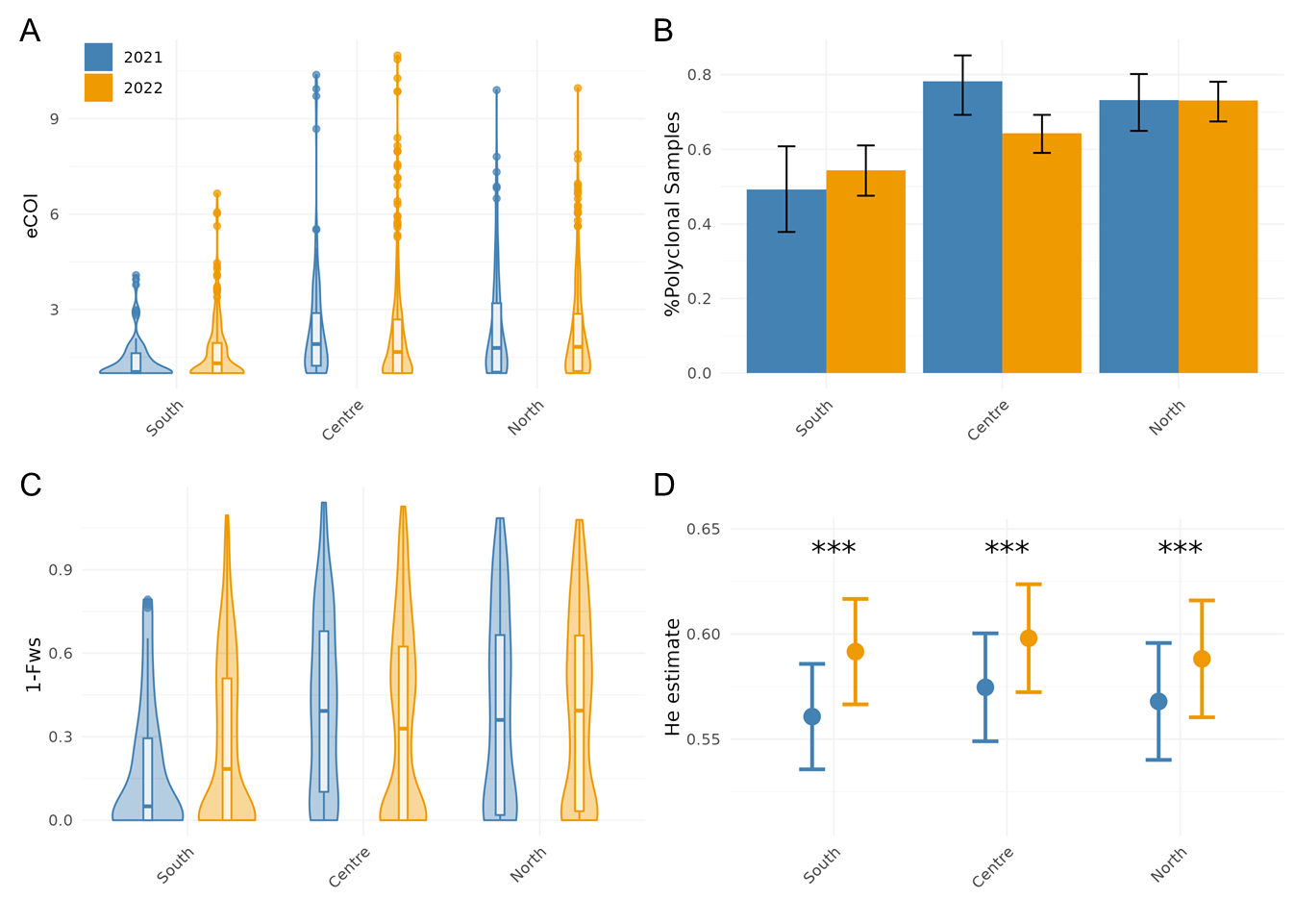
**

**Supplementary Figure 5. Relative frequency of *pfdhps* 436-437-540 haplotypes, by province.** Colors indicate administrative regions (red tones, North; green tones, Centre; blue tones, South)


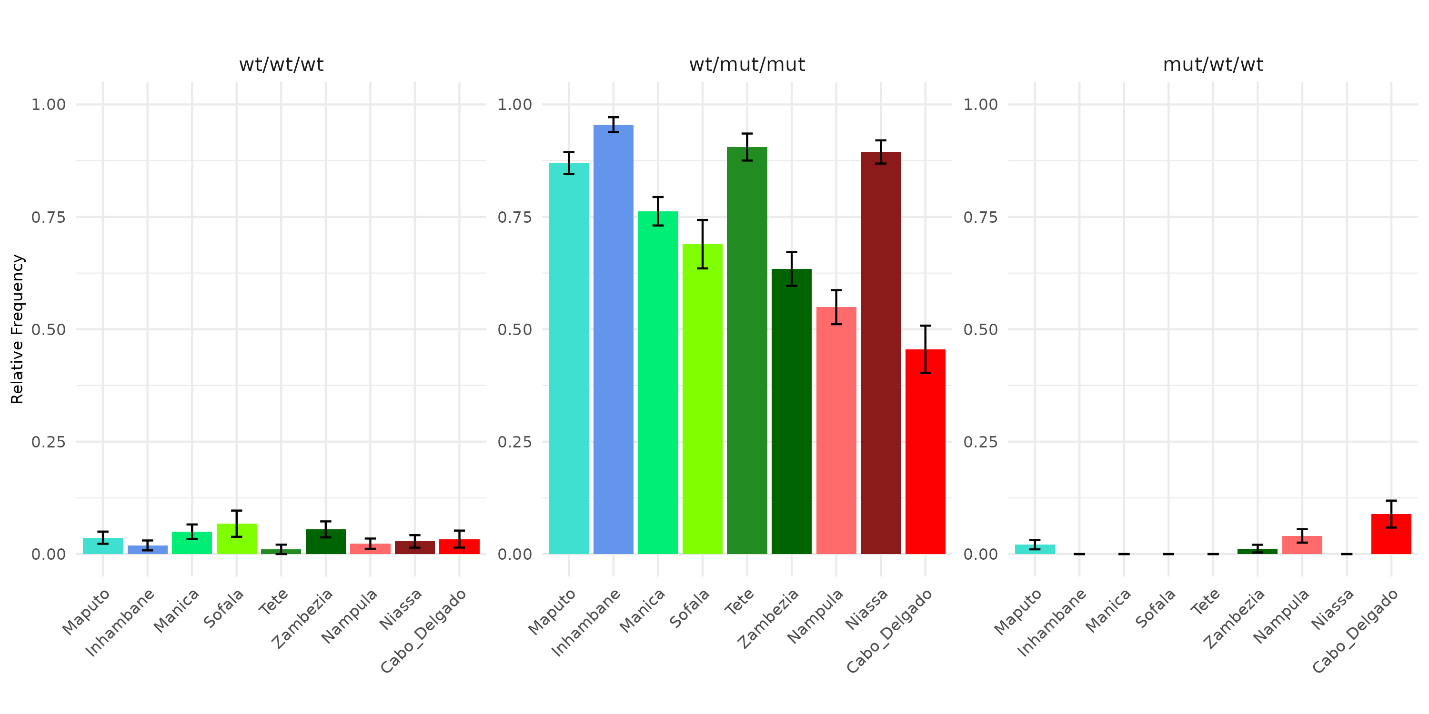


**Supplementary Figure 6. Malaria positivity rates for children under 5 year in Maputo and Manica provinces, by transmission season in 2022.** Figure shows the number of children with positive malaria rapid diagnostic tests (RDT) out of the total number of cases tested at health facilities (HF) reporting at least 96 tests done (Maputo: 21058 tests in 9 HF; Manica: 394861 tests in 75 HF). Data was obtained from the Health Information System for Monitoring and Evaluation (SIS-MA, Ministry of Health, Mozambique). Transmission season was defined as rainy (January to May) or dry (June to September).

**
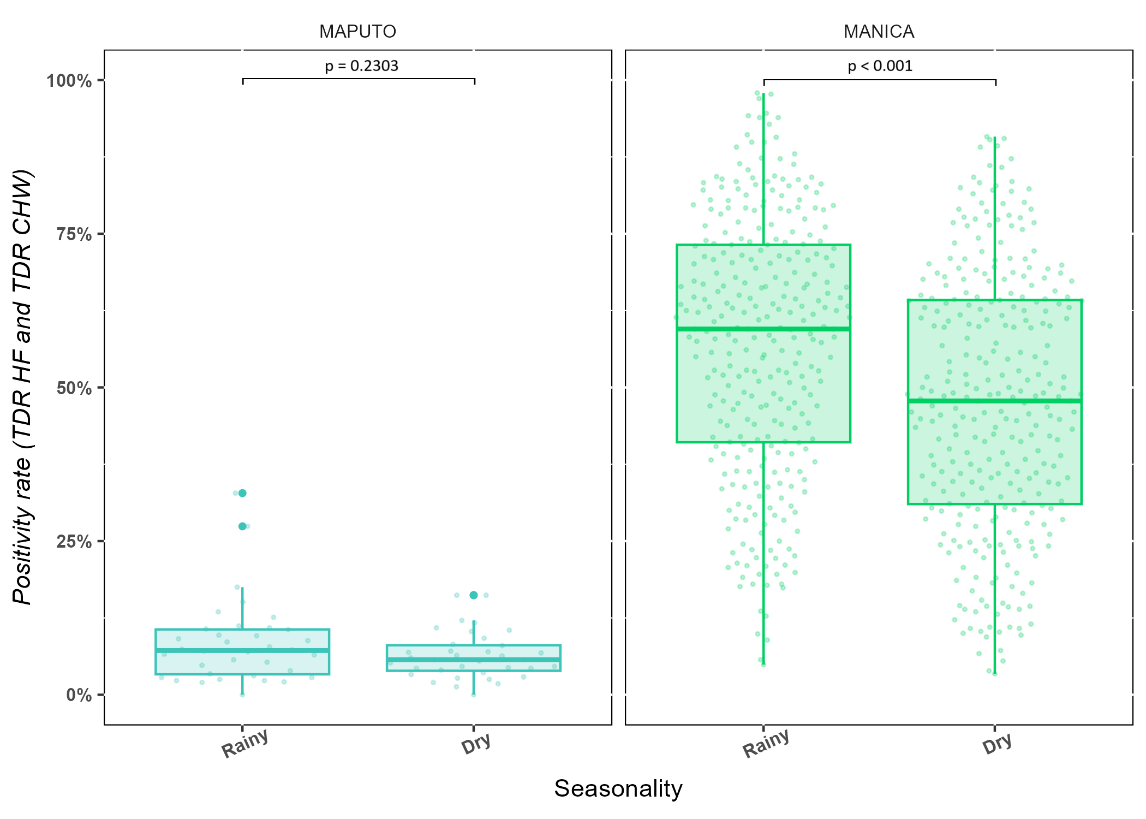
**

**Supplementary Table 1. Prevalence of antimalarial drug-resistance markers in 2021 rainy season, by province.**

|  | **Maputo** | | **Inhambane** | | **Manica** | | **Zambézia** | | **Niassa** | | **Nampula** | | |
| --- | --- | --- | --- | --- | --- | --- | --- | --- | --- | --- | --- | --- | --- |
| *pfk13* |  |  |  |  |  |  |  |  |  |  |  |  |  |
| Validated mutants | 0/4 | 0% | 0/68 | 0% | 0/26 | 0% | 0/82 | 0% | 0/51 | 0% | 0/80 | 0% |  |
| Any nsyn mutants | 0/4 | 0% | 0/64 | 0% | 0/26 | 0% | 2/78 | 2.6% | 1/49 | 2.0% | 3/76 | 4% |  |
| *pfpm2* multicopy | 0/3 | 0% | 0/55 | 0% | 0/13 | 0% | 0/56 | 0% | 0/34 | 0% | 0/57 | 0% |  |
| *pfdhfr* |  |  |  |  |  |  |  |  |  |  |  |  |  |
| N51I | 4/4 | 100% | 68/68 | 100% | 26/26 | 100% | 81/81 | 100% | 53/53 | 100% | 80/80 | 100% |  |
| C59R | 4/4 | 100% | 68/68 | 100% | 26/26 | 100% | 80/81 | 99% | 53/53 | 100% | 80/80 | 100% |  |
| S108N | 5/5 | 100% | 67/67 | 100% | 26/26 | 100% | 81/81 | 100% | 54/54 | 100% | 80/80 | 100% |  |
| I164L | 0/5 | 0% | 0/67 | 0% | 0/26 | 0% | 0/81 | 0% | 1/54 | 2% | 0/80 | 0% |  |
| triple 51-59-108 | 4/4 | 100% | 66/66 | 100% | 20/20 | 100% | 55/56 | 98% | 48/48 | 100% | 45/45 | 100% |  |
| *pfdhps* |  |  |  |  |  |  |  |  |  |  |  |  |  |
| I431V | 0/4 | 0% | 0/67 | 0% | 0/26 | 0% | 0/80 | 0% | 0/54 | 0% | 0/81 | 0% |  |
| S436A/C/F/H | 0/4 | 0% | 1/67 | 1% | 0/26 | 0% | 8/80 | 10% | 2/54 | 4% | 30/81 | 37% |  |
| A437G | 4/4 | 100% | 65/67 | 97% | 26/26 | 100% | 75/80 | 94% | 53/54 | 98% | 75/81 | 93% |  |
| K540E | 5/5 | 100% | 65/67 | 97% | 26/26 | 100% | 75/81 | 93% | 52/53 | 98% | 73/81 | 90% |  |
| A581G | 0/5 | 0% | 0/67 | 0% | 0/26 | 0% | 3/84 | 4% | 2/53 | 4% | 0/82 | 0% |  |
| A613S/T | 0/5 | 0% | 0/63 | 0% | 0/26 | 0% | 2/79 | 3% | 1/49 | 2% | 1/76 | 1% |  |
| double 437-540 | 4/4 | 100% | 64/66 | 97% | 20/20 | 100% | 51/56 | 91% | 47/48 | 98% | 39/45 | 87% |  |
| *pfdhfr/pfdhps* quintuple | 4/4 | 100% | 64/66 | 97% | 20/20 | 100% | 51/56 | 91% | 47/48 | 98% | 39/45 | 87% |  |
| *pfcrt* 72-76 CVIET | 0/4 | 0% | 0/68 | 0% | 0/26 | 0% | 0/83 | 0% | 0/54 | 0% | 0/82 | 0% |  |
| *pfmdr1* |  |  |  |  |  |  |  |  |  |  |  |  |  |
| N86Y | 0/4 | 0% | 0/67 | 0% | 0/26 | 0% | 0/82 | 0% | 0/53 | 0% | 0/80 | 0% |  |
| Y184F | 3/4 | 75% | 34/67 | 51% | 16/26 | 62% | 60/81 | 74% | 35/54 | 65% | 68/81 | 84% |  |
| D1246Y | 0/5 | 0% | 1/67 | 1% | 0/26 | 0% | 0/82 | 0% | 0/54 | 0% | 1/81 | 1% |  |

|  | **Maputo** | | **Inhambane** | | **Manica** | | **Sofala** | | **Tete** | | **Zambézia** | | **Niassa** | | **Nampula** | | **Cabo Delgado** | |  | | **Maputo (dry)** | | ***p**** | **Manica (dry)** | | ***p**** |
| --- | --- | --- | --- | --- | --- | --- | --- | --- | --- | --- | --- | --- | --- | --- | --- | --- | --- | --- | --- | --- | --- | --- | --- | --- | --- | --- |
| *pfk13* |  |  |  |  |  |  |  |  |  |  |  |  |  |  |  |  |  |  |  |  | |  |  |  |  |  |
| Validated mutants | 0/99 | 0% | 0/89 | 0% | 0/78 | 0% | 0/82 | 0% | 0/95 | 0% | 0/84 | 0% | 0/89 | 0% | 0/88 | 0% | 0/90 | 0% |  | 1/68 | | 2% | 0.226 | 0/76 | 0% | - |
| Any nsyn mutants | 2/95 | 2% | 0/89 | 0% | 1/76 | 1% | 0/81 | 0% | 1/90 | 1% | 1/81 | 1% | 3/84 | 4% | 6/87 | 7% | 0/89 | 0% |  | 4/66 | | 6% | 0.193 | 0/72 | 0% | 0.329 |
| *pfpm2* multicopy | 0/80 | 0% | 0/81 | 0% | 0/59 | 0% | 0/69 | 0% | 0/88 | 0% | 0/58 | 0% | 0/59 | 0% | 0/52 | 0% | 0/90 | 0% |  | 0/57 | | 0% | - | 0/65 | 0% | - |
| *pfdhfr* |  |  |  |  |  |  |  |  |  |  |  |  |  |  |  |  |  |  |  |  | |  |  |  |  |  |
| N51I | 100/100 | 100% | 89/90 | 99% | 77/78 | 99% | 81/83 | 98% | 94/95 | 99% | 84/85 | 99% | 90/90 | 100% | 91/91 | 100% | 89/90 | 99% |  | 69/69 | | 100% | - | 76/78 | 97% | 0.560 |
| C59R | 100/100 | 100% | 90/90 | 100% | 77/78 | 99% | 83/83 | 100% | 91/95 | 96% | 84/85 | 99% | 90/90 | 100% | 91/91 | 100% | 90/90 | 100% |  | 69/69 | | 100% | - | 76/78 | 97% | 0.560 |
| S108N | 100/100 | 100% | 90/90 | 100% | 79/79 | 100% | 84/84 | 100% | 96/96 | 100% | 83/84 | 99% | 90/90 | 100% | 92/92 | 100% | 90/90 | 100% |  | 70/70 | | 100% | - | 78/78 | 100% | - |
| I164L | 0/100 | 0% | 0/90 | 0% | 0/79 | 0% | 0/84 | 0% | 0/96 | 0% | 0/84 | 0% | 0/90 | 0% | 0/92 | 0% | 0/90 | 0% |  | 0/70 | | 0% | - | 0/78 | 0% | - |
| triple 51-59-108 | 94/94 | 100% | 87/88 | 99% | 56/59 | 95% | 66/67 | 99% | 85/90 | 94% | 52/54 | 96% | 83/83 | 100% | 67/67 | 100% | 55/56 | 98% |  | 62/62 | | 100% | - | 60/64 | 94% | 0.780 |
| *pfdhps* |  |  |  |  |  |  |  |  |  |  |  |  |  |  |  |  |  |  |  |  | |  |  |  |  |  |
| I431V | 0/100 | 0% | 0/89 | 0% | 0/79 | 0% | 0/84 | 0% | 0/96 | 0% | 0/84 | 0% | 0/89 | 0% | 0/92 | 0% | 0/90 | 0% |  | 0/70 | | 0% | - | 0/78 | 0% | - |
| S436A/C/F/H | 4/100 | 4% | 0/89 | 0% | 0/79 | 0% | 1/84 | 1% | 2/96 | 2% | 5/84 | 6% | 5/89 | 6% | 26/92 | 28% | 35/90 | 39% |  | 4/70 | | 6% | 0.603 | 0/78 | 0% | - |
| A437G | 95/100 | 95% | 88/89 | 99% | 72/79 | 91% | 76/84 | 90% | 95/96 | 99% | 78/84 | 93% | 86/89 | 97% | 89/92 | 97% | 78/90 | 87% |  | 66/70 | | 94% | 0.838 | 76/78 | 97% | 0.090 |
| K540E | 93/100 | 93% | 88/89 | 99% | 72/79 | 91% | 76/83 | 92% | 93/95 | 98% | 78/84 | 93% | 85/89 | 96% | 88/92 | 96% | 78/90 | 87% |  | 65/69 | | 94% | 0.755 | 74/78 | 95% | 0.360 |
| A581G | 1/100 | 1% | 0/89 | 0% | 0/79 | 0% | 0/83 | 0% | 1/95 | 1% | 2/84 | 2% | 2/89 | 2% | 0/92 | 0% | 0/90 | 0% |  | 0/70 | | 0% | 0.401 | 0/78 | 0% | - |
| A613S/T | 2/96 | 2% | 0/88 | 0% | 0/75 | 0% | 0/80 | 0% | 0/90 | 0% | 2/82 | 2% | 1/82 | 1% | 4/91 | 4% | 5/88 | 6% |  | 0/69 | | 0% | 0.228 | 0/73 | 0% | - |
| double 437-540 | 88/94 | 94% | 87/88 | 99% | 52/59 | 88% | 58/67 | 87% | 88/90 | 98% | 49/54 | 91% | 79/83 | 95% | 64/67 | 96% | 44/56 | 79% |  | 58/62 | | 94% | 0.986 | 62/64 | 97% | 0,063 |
| *pfdhfr/pfdhps* quintuple | 88/94 | 94% | 86/88 | 98% | 49/59 | 83% | 58/67 | 87% | 83/90 | 92% | 47/54 | 87% | 79/83 | 95% | 64/67 | 96% | 43/56 | 77% |  | 58/62 | | 94% | 0.986 | 58/64 | 91% | 0.212 |
| *pfcrt* 72-76 CVIET | 0/100 | 0% | 0/89 | 0% | 0/78 | 0% | 0/84 | 0% | 0/97 | 0% | 0/83 | 0% | 0/90 | 0% | 1/93 | 1% | 0/90 | 0% |  | 0/70 | | 0% | - | 0/77 | 0% | - |
| *pfmdr1* |  |  |  |  |  |  |  |  |  |  |  |  |  |  |  |  |  |  |  |  | |  |  |  |  |  |
| N86Y | 0/100 | 0% | 0/90 | 0% | 0/78 | 0% | 0/84 | 0% | 0/96 | 0% | 0/84 | 0% | 0/89 | 0% | 1/91 | 1% | 0/89 | 0% |  | 0/70 | | 0% | - | 0/74 | 0% | - |
| Y184F | 54/100 | 54% | 60/90 | 67% | 52/79 | 66% | 64/84 | 76% | 65/96 | 68% | 61/84 | 73% | 74/89 | 83% | 68/91 | 75% | 67/90 | 74% |  | 52/70 | | 74% | 0.007 | 50/76 | 66% | 0.997 |
| D1246Y | 0/100 | 0% | 1/88 | 1% | 0/80 | 0% | 0/83 | 0% | 0/97 | 0% | 0/85 | 0% | 0/90 | 0% | 0/93 | 0% | 0/90 | 0% |  | 0/70 | | 0% | - | 2/76 | 3% | - |
| *as compared to the same province in the raiy season | | | | |  |  |  |  |  |  |  |  |  |  |  |  |  |  |  |  | |  |  |  |  |  |

**Supplementary Table 2. Prevalence of antimalarial drug-resistance markers in 2022, by province.**

**Supplementary Table 3. Multivariable logistic regression model for** **factors associated with drug -resistance markers in 2021.**

| **2021** | ***pfk13* n-syn** (N=137) | | |  | ***dhfr-dhps* quintuple** (N=239) | | |  | ***pfdhps*-S436A/C/F/H** (N=312) | | |  | ***pfdhps*-A581G** (N=245) | | |  | ***pfmdr1-*Y184F** (N=313) | | |
| --- | --- | --- | --- | --- | --- | --- | --- | --- | --- | --- | --- | --- | --- | --- | --- | --- | --- | --- | --- |
|  | OR | (95%CI) | p-value |  | OR | (95%CI) | p-value |  | OR | (95%CI) | p-value |  | OR | (95%CI) | p-value |  | OR | (95%CI) | p-value |
| **Region** |  |  |  |  |  |  |  |  |  |  |  |  |  |  |  |  |  |  |  |
| South | 1 |  |  |  | 1 |  |  |  | 1 |  |  |  | 1 |  |  |  | 1 |  |  |
| Centre | 0.4 | (0.8, 2.6) | 0.360 |  | 0.3 | (0.1, 1.9) | 0.224 |  | 6.3 | (0.8, 52.9) | 0.086 |  | 1.7 | (0.3, 10.4) | 0.578 |  | 2.1 | (1.1, 4.0) | **0.026** |
| North | - | - | - |  | 0.4 | (0.1, 1.8) | 0.213 |  | 23.0 | (3.0, 174.2) | **0.002** |  | - | - | - |  | 2.9 | (1.5, 5.4) | **0.001** |
| **Age** |  |  |  |  |  |  |  |  |  |  |  |  |  |  |  |  |  |  |  |
| <5 yr | 1 |  |  |  | 1 |  |  |  | 1 |  |  |  | 1 |  |  |  | 1 |  |  |
| ≥5 yr | - | - | - |  | 1.2 | (0.4, 3.6) | 0.794 |  | 0.9 | (0.5, 1.8) | 0.796 |  | 1.2 | (0.2, 7.4) | 0.860 |  | 0.9 | (0.6, 1.5) | 0.752 |
| **Gender** |  |  |  |  |  |  |  |  |  |  |  |  |  |  |  |  |  |  |  |
| Female | 1 |  |  |  | 1 |  |  |  | 1 |  |  |  | 1 |  |  |  | 1 |  |  |
| Male | 0.2 | (0.02, 1.9) | 0.166 |  | 0.4 | (0.1, 1.2) | 0.097 |  | 1.4 | (0.7, 2.8) | 0.341 |  | 0.7 | (0.1, 4.6) | 0.740 |  | 0.7 | (0.4, 1.1) | 0.138 |
| **Parasitemia** |  |  |  |  |  |  |  |  |  |  |  |  |  |  |  |  |  |  |  |
| <500 p/uL | 1 |  |  |  | 1 |  |  |  | 1 |  |  |  | 1 |  |  |  | 1 |  |  |
| ≥500 p/uL | 0.8 | (0.1, 4.81) | 0.831 |  | 0.6 | (0.2, 2.4) | 0.516 |  | 2.1 | (0.9, 5.0) | 0.109 |  | 0.3 | (0.04, 1.8) | 0.175 |  | 1.0 | (0.6, 1.8) | 0.930 |

**Supplementary Table 4. Multivariable logistic regression model for** **factors associated with drug-resistance markers in 2022.**

| **2022** | ***pfk13* n-syn** (N=761) | | |  | ***dhfr-dhps* quintuple** (N=658) | | |  | ***pfdhps*-S436A/C/F/H** (N=792) | | |  | ***pfdhps*-A581G** (N=790) | | |  | ***pfmdr1-*Y184F** (N=803) | | |
| --- | --- | --- | --- | --- | --- | --- | --- | --- | --- | --- | --- | --- | --- | --- | --- | --- | --- | --- | --- |
|  | OR | (95%CI) | p-value |  | OR | (95%CI) | p-value |  | OR | (95%CI) | p-value |  | OR | (95%CI) | p-value |  | OR | (95%CI) | p-value |
| **Region** |  |  |  |  |  |  |  |  |  |  |  |  |  |  |  |  |  |  |  |
| South | 1 |  |  |  | 1 |  |  |  | 1 |  |  |  | 1 |  |  |  | 1 |  |  |
| Centre | 0.6 | (0.1, 4.6) | 0.653 |  | 0.4 | (0.2, 0.8) | **0.019** |  | 0.7 | (0.2, 2.3) | 0.537 |  | 1.9 | (0.2, 19.8) | 0.581 |  | 1.7 | (1.1, 2.5) | **0.012** |
| North | 1.7 | (0.3, 10.3) | 0.551 |  | 0.5 | (0.2, 1.2) | 0.144 |  | 7.7 | (2.6, 22.6) | **<0.001** |  | 1.8 | (0.1, 23.6) | 0.664 |  | 2.4 | (1.6, 3.8) | **<0.001** |
| **Age** |  |  |  |  |  |  |  |  |  |  |  |  |  |  |  |  |  |  |  |
| <5 yr | 1 |  |  |  | 1 |  |  |  | 1 |  |  |  | 1 |  |  |  | 1 |  |  |
| ≥5 yr | 0.5 | (0.1, 2.0) | 0.333 |  | 1.3 | (0.7, 2.3) | 0.443 |  | 0.24 | (0.1, 0.6) | **0.001** |  | 1.8 | (0.3, 9.7) | 0.488 |  | 1.1 | 0.8, 1.5 | 0.752 |
| **Gender** |  |  |  |  |  |  |  |  |  |  |  |  |  |  |  |  |  |  |  |
| Female | 1 |  |  |  | 1 |  |  |  | 1 |  |  |  | 1 |  |  |  | 1 |  |  |
| Male | 0.5 | (0.1, 1.5) | 0.195 |  | 0.6 | (0.4, 1.1) | 0.098 |  | 1.1 | (0.6, 1.8) | 0.793 |  | 4.7 | (0.6, 40.8) | 0.157 |  | 0.9 | 0.7, 1.2 | 0.587 |
| **Parasitemia** |  |  |  |  |  |  |  |  |  |  |  |  |  |  |  |  |  |  |  |
| <500 p/uL | 1 |  |  |  | 1 |  |  |  | 1 |  |  |  | 1 |  |  |  | 1 |  |  |
| ≥500 p/uL | 0.02 | (0.01, 0.1) | **<0.001** |  | 1.3 | (0.6, 2.5) | 0.529 |  | 0.9 | (0.5, 1.8) | 0.875 |  | 1.1 | (0.1, 9.8) | 0.926 |  | 1.1 | 0.7, 1.6 | 0.758 |

**Supplementary Table 5. Multivariable logistic regression model for** **factors associated with genetic diversity metrics in 2022.**

|  | **eCOI** (N=137) | | |  | **% polyclonal** (N=239) | | |  | **1-Fws** (N=312) | | |
| --- | --- | --- | --- | --- | --- | --- | --- | --- | --- | --- | --- |
|  | Coef. | (95%CI) | p-value |  | OR | (95%CI) | p-value |  | Coef. | (95%CI) | p-value |
| **Region** |  |  |  |  |  |  |  |  |  |  |  |
| South | 1 |  |  |  | 1 |  |  |  | 1 |  |  |
| Centre | 0.5 | (0.2, 0.8) | **0.001** |  | 1.4 | (0.9, 2.0) | 0.115 |  | 0.1 | (0.03, 0.2) | **0.003** |
| North | 0.6 | (0.3, 0.9) | **<0.001** |  | 2.0 | (1.3, 3.1) | **0.002** |  | 0.1 | (0.06, 0.2) | **<0.001** |
| **Age** |  |  |  |  |  |  |  |  |  |  |  |
| <5 yr | 1 |  |  |  | 1 |  |  |  | 1 |  |  |
| ≥5 yr | -0.05 | (-0.3, 0.2) | 0.693 |  | 0.8 | (0.6, 1.1) | 0.264 |  | -0.01 | (-0.1, 0.04) | 0.597 |
| **Gender** |  |  |  |  |  |  |  |  |  |  |  |
| Female | 1 |  |  |  | 1 |  |  |  | 1 |  |  |
| Male | 0.08 | (-0.1, 0.3) | 0.451 |  | 1.1 | (0.8, 1.5) | 0.483 |  | -0.001 | (-0.04, 0.04) | 0.969 |
| **Parasitemia** |  |  |  |  |  |  |  |  |  |  |  |
| <500 p/uL | 1 |  |  |  | 1 |  |  |  | 1 |  |  |
| ≥500 p/uL | 0.2 | (-0.1, 0.5) | 0.110 |  | 0.9 | (0.6, 1.5) | 0.865 |  | 0.04 | (-0.2, 0.1) | 0.157 |

**Supplementary Table 6. Multivariable logistic regression model for** **factors associated with drug-resistance markers in the provinces of Maputo and Manica in 2022, including seasonality.** There were insufficient observations to build models for *pfk13*-non synonymous and *pfdhps-*436 variants in Manica province, and for *pfdhps*-581 in both provinces.

|  |  | **Maputo** | | | | | | | | | | | | | | |  | | **Manica** | | | | | | |
| --- | --- | --- | --- | --- | --- | --- | --- | --- | --- | --- | --- | --- | --- | --- | --- | --- | --- | --- | --- | --- | --- | --- | --- | --- | --- |
|  | ***pfk13* n-syn** (N=160) | | |  | ***dhfr-dhps* quintuple** (N=137) | | |  | ***pfdhps*-S436A/C/F/H** (N=169) | | |  | ***pfmdr1-*Y184F** (N=169) | | |  | | ***dhfr-dhps* quintuple** (N=155) | | | |  | ***pfmdr1-*Y184F** (N=155) | | |
|  | OR | (95%CI) | p-value |  | OR | (95%CI) | p-value |  | OR | (95%CI) | p-value |  | OR | (95%CI) | p-value |  | | OR | | (95%CI) | p-value |  | OR | (95%CI) | p-value |
| **Season** |  |  |  |  |  |  |  |  |  |  |  |  |  |  |  |  | |  | |  |  |  |  |  |  |
| Rainy | 1 |  |  |  | 1 |  |  |  | 1 |  |  |  | 1 |  |  |  | | 1 | |  |  |  | 1 |  |  |
| Dry | 10.5 | (1.1, 99.1) | **0.040** |  | 1.5 | (1.7, 29.9) | 0.594 |  | 1.5 | (0.3, 6.4) | 0.594 |  | 2.4 | (1.2, 4.7) | **0.010** |  | | 2.1 | | (0.7, 6.5) | 0.210 |  | 1 | (0.5, 1.9) | 0.991 |
| **Age** |  |  |  |  |  |  |  |  |  |  |  |  |  |  |  |  | |  | |  |  |  |  |  |  |
| <5 yr | 1 |  |  |  | 1 |  |  |  | 1 |  |  |  | 1 |  |  |  | | 1 | |  |  |  | 1 |  |  |
| ≥5 yr | 0.3 | (0.01, 5.4) | 0.384 |  | 7.1 | (1.7, 29.9) | **0.007** |  | 1.1 | (0.2, 6.0) | 0.909 |  | 1.1 | (0.5, 2.3) | 0.795 |  | | 1.1 | | (0.3, 3.2) | 0.908 |  | 1.1 | (0.6, 2.2) | 0.712 |
| **Gender** |  |  |  |  |  |  |  |  |  |  |  |  |  |  |  |  | |  | |  |  |  |  |  |  |
| Female | 1 |  |  |  | 1 |  |  |  | 1 |  |  |  | 1 |  |  |  | | 1 | |  |  |  | 1 |  |  |
| Male | 0.3 | (0.03, 2.6) | 0.276 |  | 0.6 | (0.15, 2.4) | 0.478 |  | 7.3 | (0.9, 61.1) | 0.067 |  | 1.1 | (0.6, 2.2) | 0.690 |  | | 0.2 | | (0.05, 0.7) | **0.015** |  | 0.9 | (0.5, 1.7) | 0.728 |
| **Parasitemia** |  |  |  |  |  |  |  |  |  |  |  |  |  |  |  |  | |  | |  |  |  |  |  |  |
| <500 p/uL | 1 |  |  |  | 1 |  |  |  | 1 |  |  |  | 1 |  |  |  | | 1 | |  |  |  | 1 |  |  |
| ≥500 p/uL | 0.01 | (0.001, 0.2) | **0.002** |  | - | - | - |  | 0.4 | (0.1, 2.5) | 0.359 |  | 1.1 | (0.4, 2.7) | 0.832 |  | | 0.3 | | (0.03, 2.3) | 0.234 |  | 0.9 | (0.4, 2.4) | 0.876 |
